# A Deep Mining Strategy for Peptide Rapid Identification in *Lactobacillus reuteri* Based on LC–MS/MS Integrated with FBMN and De Novo Sequencing

**DOI:** 10.3390/metabo14090467

**Published:** 2024-08-23

**Authors:** Yilang Zuo, Shilin Gong, Li Zhang, Jie Zhou, Jian-Lin Wu, Na Li

**Affiliations:** State Key Laboratory of Quality Research in Chinese Medicine, Macau Institute for Applied Research in Medicine and Health, Macau University of Science and Technology, Taipa, Macau 999078, China; 2009853vct20002@student.must.edu.mo (Y.Z.); 21098532ct30001@student.must.edu.mo (S.G.); 2109853qct30001@student.must.edu.mo (L.Z.); 2009853zct20001@student.must.edu.mo (J.Z.)

**Keywords:** *Lactobacillus reuteri*, peptides, cyclic dipeptides, feature-based molecular networking, molecular docking, anti-inflammatory

## Abstract

*Lactobacillus reuteri* (*L. reuteri*) is widely recognized as a probiotic that produces prebiotics. However, studies on bioactive peptides or amino acid (AA) derivatives produced by *L. reuteri* are still lacking, whereas many bioactive peptides and AA derivatives have been found in other *Lactobacillus* species. In addition, rapid identification of peptides is challenged by the large amount of data and is limited by the coverage of protein databases. In this study, we performed a rapid and thorough profile of peptides in *L. reuteri* incorporating Global Natural Products Social Molecular Networking (GNPS) platform database searching, de novo sequencing, and deep mining, based on feature-based molecular networking (FBMN). According to FBMN, it was found that peptides containing identical or similar AA compositions were grouped into the same clusters, especially cyclic dipeptides (CDPs). Therefore, the grouping characteristics of clusters, differences in precursor ions, and characteristic fragment ions were utilized for the mining of deeply unknown compounds. Through this strategy, a total of 192 compounds, including 184 peptides, were rapidly identified. Among them, 53 CDPs, including four novel ones, were found for the first time in *L. reuteri*. Then, one of the novel CDPs, cyclo(5-OMe-Glu-4-OH-Pro), was isolated and characterized, which was consistent with the identification results. Moreover, some of the identified peptides exhibited considerable interactions with seven anti-inflammatory-related target proteins through molecular docking. According to the binding energies of peptides with different AA consistencies, it was considered that the existence of unnatural AAs in CDPs might contribute to their anti-inflammatory activity. These results provide a valuable strategy for the rapid identification of peptides, including CDPs. This study also reveals the substance basis for the potential anti-inflammatory effects exerted by *L. reuteri*.

## 1. Introduction

Prebiotics produced by probiotics are microbiota management tools for improving host health [1]. Therefore, the investigation of prebiotics is meaningful for the application of probiotics and the development of new drugs. *Lactobacillus reuteri* (*L. reuteri*) is a probiotic known to have a variety of biological functions and is involved in human physiological activities, such as those of the immune [2], digestive [3], and urinary systems [4]. For the prebiotics produced by *L. reuteri*, such as reuterin [5], indole-3-lactic acid [6,7], and organic acid [8], there have been studies on their beneficial effects related to anti-colorectal cancer, anti-inflammatory, or antibacterial properties. It is worth noting that studies have shown *L. reuteri* can alleviate inflammatory bowel disease [6,9]. Despite these findings, research into the active peptides or AA derivatives produced by *L. reuteri* remains limited. In contrast, other *Lactobacillus* species have been reported to generate numerous bioactive peptides and AA derivatives [10,11,12]. For instance, *Lactobacillus helveticus* was reported to produce hypotensive peptides [13], *Lactobacillus fermentu* could generate antibacterial peptides [14], and *Lactobacillus rhamnosus* contained anti-inflammatory and antibiotic peptides [15]. However, only several bioactive peptides were reported in *L. reuteri*, such as AP48-MapA [16], ORF-1 [17], and three cyclic dipeptides (CDPs) [18]; there are few bioactive peptides reported in this strain. Given the diverse range of bioactive peptides produced by other *Lactobacillus* species, and considering the limited number of reported peptides produced by *L. reuteri*, it is reasonable to speculate that many more bioactive peptides and AA derivatives remain undiscovered in *L. reuteri*. Therefore, a comprehensive investigation of these compounds in *L. reuteri* is necessary for bioactive peptide exploration.

Presently, rapid identification of peptides is challenged by the analysis of large amounts of data and is limited to identifying the peptides beyond protein databases [19]. Due to the high sensitivity and ability to rapidly detect compounds in complex matrices, liquid chromatography–mass spectrometry (LC–MS) has been widely used for peptidomics analysis and identification [20]. Currently, there are many analytical methods for the rapid identification of compounds, such as feature-based molecular networking (FBMN) [21]. This strategy has been reported to allow rapid identification of known peptides and suggest new peptides with similar structures [22]. In addition, proteomics analysis software, such as PEAKS studio, is also efficient for peptide identification. To rapidly and comprehensively identify the compounds in *L. reuteri*, a combination of multiple analytical methods was attempted to deeply mine the information in MS data for peptide discovery.

In this study, the aim was to perform a rapid and thorough profile of compounds from *L. reuteri* by incorporating several analytical methods based on FBMN. A total of 192 compounds were identified, including 184 peptides. Among them, 53 CDPs were found for the first time in *L. reuteri*, four of which were novel CDPs. Next, structural characterization of the new compound was carried out to prove the feasibility of the method. Moreover, the identified peptides were predicted to have potential anti-inflammatory activity by molecular docking.

## 2. Materials and Methods

### 2.1. Materials and Reagents

Acetonitrile (ACN, HPLC grade), methanol (MeOH, HPLC grade), ethyl acetate (EtOAc, ACS grade), *n*-butyl alcohol (*n*-BuOH, ACS grade), methylene chloride (CH_2_Cl_2_, ACS grade), *n*-hexane (ACS grade), and acetone (ACS grade) were obtained from Anaqua Chemicals Supply Inc., Ltd. (Houston, TA, USA). MS-grade formic acid and ammonium hydroxide were provided by Sigma-Aldrich Laboratories, Inc. (St. Louis, MO, USA). Deionized water (Milli-Q-Water) was prepared using a Millipore water purification system (Merck, Hong Kong, China). Methanol-*d*_4_ (D ≥ 99.8%) was purchased from Thermo Fisher Scientific Inc. (Waltham, MA, USA).

### 2.2. General Experimental Procedures

The samples were determined by an Agilent 1290 Infinity LC system (Agilent, Santa Clara, CA, USA). Mass spectrometry was conducted on an Agilent 6550 UHD accurate-mass Q-TOF/MS system with a dual jet stream electrospray ion source (dual AJS ESI). The semipreparative HPLC separations were performed with an Agilent 1100 multiple-wavelength absorbance detector with a mobile phase using the H_2_O/ACN system. All NMR data were collected by Bruker Ascend 600 NMR (Bruker, Billerica, MA, USA) at 600 MHz for ^1^H NMR and 150 MHz for ^13^C NMR.

### 2.3. Bacterial Strain and Growth Conditions

The *L. reuteri* strain was purchased from the China General Microbiological Culture Collection Center. *L. reuteri* was cultivated in Man-Rogosa-Sharpe (MRS) medium for 48 h at 37 °C in an anaerobic chamber (Forma Scientific Company, Marietta, GA, USA, containing an atmosphere of 85% N_2_, 10% H_2_O, and 5% CO_2_). The MRS components included glucose 20 g/L, peptone 10 g/L, beef extract 10 g/L, yeast extract 5 g/L, dipotassium phosphate 2 g/L, magnesium sulfate heptahydrate 0.2 g/L, manganese sulfate monohydrate 0.05 g/L, anhydrous sodium acetate 5 g/L, and triammonium hydrogen citrate 2 g/L. Some of the MRS in conical bottles were set as controls without any bacterial inoculation.

### 2.4. Extraction and Isolation

The bacterial samples were centrifuged to obtain the cell-free supernatant. *L. reuteri* cell-free supernatant (8.4 L) was extracted at room temperature using EtOAc (30.8 L) and *n*-BuOH (19.6 L) successively. After rotary evaporation and nitrogen drying, the obtained *n*-butanol extract (36.7 g) was subjected to preliminary separation via silica gel column chromatography and eluted with CH_2_Cl_2_ with a gradually increasing gradient of CH_3_OH. The controls underwent the same operation.

For the isolation of cyclo(5-OMe-Glu-4-OH-Pro), eleven fractions (F-A to F-K) were grouped and pooled together according to TLC profiling. Fraction F (0.9 g) was subjected to silica gel using CH_2_Cl_2_/CH_3_OH as the eluent to produce 52 fractions. Fraction 20–22 (53.2 mg) was purified by semipreparative HPLC with ACN in water (5%, *v*/*v*, 20 min, 3 mL/min) and an Xbridge BEH Prep C18 column (250 mm × 10.0 mm, 5 μm, Waters) to give cyclo(5-OMe-Glu-4-OH-Pro) (1.0 mg).

### 2.5. Sample Preparation

The collected fractions and controls (without bacterial inoculation) from preliminary separation were partially dissolved in MeOH. After centrifugation at 13,500 rpm for 5 min, the supernatants were transferred to HPLC vials for LC–MS analysis.

For solid-phase extraction (MCX-SPE) samples, the crude extract fractions and control (MRS) were centrifuged at 10,000× *g* for 15 min at room temperature. The supernatant was nitrogen blown dry and dissolved in 1 mL of 2% formic acid solution and shaken for 5 min on a Mixer Vortex (Thermo Scientific Inc., Waltham, MA, USA). Sample pretreatment was performed according to the previous method for SPE peptide enrichment [22]. The Oasis MCX SPE cartridges (3 cc, 60 mg, 60 μm, Waters, Milford, MA, USA) were pretreated sequentially with 2 mL of MeOH, 2 mL of H_2_O, and 1 mL of 2% formic acid solution. Then, the samples were loaded into cartridges and flushed sequentially with 1.5 mL of 2% formic acid solution and 1.5 mL of MeOH. Next, the enriched peptides were eluted with 1.5 mL of 2% ammonium hydroxide dissolved in MeOH under gravity. Finally, the samples were dried with nitrogen at room temperature and reconstituted in 200 μL MeOH/water (50/50, *v*/*v*). After centrifugation at 13,500 rpm for 5 min, the supernatant was transferred to an HPLC vial for LC–MS analysis.

### 2.6. Mass Spectral Data Acquisition

A Waters ACQUITY UPLC HSS T3 column (2.1 mm × 100 mm, 1.8 μm) with 0.1% formic acid in water (A) and ACN (B) was used. Two solvents were eluted at a flow rate of 0.3 mL/min according to the following gradient: 0.0–0.5 min, 5.0% B; 0.5–22.0 min, 5.0–27.0% B; 22.0–23.0 min, 27.0–95.0% B; and 23.0–24.9 min, 95.0% B.

Positive ESI ion mode was applied, and the parameters were set as follows: sheath gas temperature at 300 °C, sheath gas flow at 11 L/min, dry gas temperature at 250 °C, dry gas flow at 15 L/min, nebulizer at 22 psi, and capillary voltage at 3500 V. Acquisition *m*/*z* ranges were set as 100–1700 for MS analysis and 50–1700 for AutoMS2 analysis. External calibration with a low flow of TOF reference mixture was performed prior to data acquisition, and hexakis-(1H,1H,3H-tetrafluoropropoxy) phosphazene *m*/*z* 922.0098 (C_18_H_18_N_3_O_6_P_3_F_24_) was used as a lock mass internal calibrant during data acquisition (Agilent Technologies).

### 2.7. MZmine 2.53 Data-Preprocessing Parameters

The raw data were ingested and converted to mzML file format using MSConvert software (http://proteowizard.sourceforge.net/, accessed on 19 July 2024) [23]. Subsequently, the mzML files were processed using MZmine 2.53 software [24]. Mass detection was achieved by using the center mass detector with the noise level set to 1.0 × 10^3^ for the MS1 noise level and 1.0 × 10^2^ for the MS2 noise level. Chromatogram building was generated using ADAP (Automated Data Analysis Pipeline) chromatogram builder with a minimum scan group size of 5, a minimum group intensity threshold of 3.0 × 10^3^, a minimum highest intensity of 3.0 × 10^3^, and an *m*/*z* tolerance of 0.01 or 20 ppm. The local minimum search deconvolution algorithm was used with the following settings: chromatographic threshold = 10%, minimum retention time range = 0.25 min, minimum relative height = 10%, minimum absolute height = 1.0 × 10^3^, minimum ratio of peak top/edge = 1, and peak duration range = 0.00–3.00 min. Chromatograms were deisotoped utilizing the isotopic peaks grouper algorithm with an *m*/*z* tolerance of 0.01 (or 20 ppm), an RT tolerance of 0.25, and a maximum charge of 1, and the representative isotope used was the most intense. The parameter settings of the join aligner were as follows: *m*/*z* tolerance of 0.01 (or 20 ppm), weight for *m*/*z* of 75, RT tolerance of 0.25 min, and weight for RT of 25. The feature list rows filter parameters were set according to minimum peaks in a row of 2, minimum peaks in an isotope pattern of 1 and keeping only peaks with MS2 scan of the Global Natural Products Social Molecular Networking platform (GNPS). Next, the peak list was eventually gap-filled with the peak finder module: intensity tolerance of 10%, *m*/*z* tolerance of 0.01 (or 20 ppm), and RT tolerance of 0.25. After data processing, two files were exported for GNPS: an mgf file and an associated quantitative peak area table (csv file).

### 2.8. Feature-Based Molecular Networking

The mgf file and csv file processed by Mzmine 2.53 were uploaded to the GNPS platform (https://gnps.gucsd.edu/, accessed on 19 July 2024) [25]. Molecular networks were constructed using the characteristic molecular networks of the GNPS platform. After running the spectral clustering algorithm, the Cytoscape data were downloaded from the job status page (https://gnps.ucsd.edu/ProteoSAFe/status.jsp?task=70bf4439a07745289962b72780a3ca0e/, accessed on 19 July 2024) and then imported into Cytoscape (version 3.10.0) (https://cytoscape.org/, accessed on 19 July 2024) to create a network visualization [26].

### 2.9. Identification of Peptides Using PEAKS Studio

The MS/MS raw data were analyzed using PEAKS studio version 10.5 (Bioinformatics Solutions Inc., Waterloo, ON, Canada). Searches were performed utilizing the SwissProt database and de novo sequencing. The search parameters were set as follows: non-enzyme (unspecific), precursor ion mass error tolerance at 10 ppm, fragment ion error tolerance at 0.02 Da, dynamic modifications: none and monoisotopic precursor mass. To reduce false positives of identified peptides, the peptide hit threshold (−10 logP) was set at ≥15, and the de novo score (average local confidence, ALC%) threshold was set to 50%.

### 2.10. In Silico Analysis

The potential activity and anti-inflammatory activities of the sequence-based peptides were predicted using PeptideRanker (http://distilldeep.ucd.ie/PeptideRanker/, accessed on 19 July 2024) [27] and AIPpred tool (http://kurata14.bio.kyutech.ac.jp/PreAIP/, accessed on 19 July 2024) [28]. In PeptideRanker, the closer the predicted probability value was to 1, the higher the possibility of the peptide having biological activity. In AIPpred, the score range at 0.342–0.388 was low confidence AIP value, at 0.388–0.468 was medium confidence AIP value, and higher than 0.468 was high confidence AIP value.

### 2.11. Molecular Docking

The crystal structures of NIMA-related kinase 7 (NEK7; PDB ID: 2WQN), cathepsin C (Cat C; PDB ID: 4CDE), gasdermin D (GSDMD; PDB ID: 5WQT), the human TLR4-human MD-2-*E. coli* LPS Ra complex (TLR4/MD2; PDB ID: 3FXI), the tumor necrosis factor-alpha (TNF-α; PDB ID: 1TNF), the extra-cellular domains of the human interleukin-6 receptor alpha chain (IL-6; PDB ID: 1N26), and the interleukin-1 receptor complex (IL-1β; PDB ID: 3O4O) were downloaded from the Protein Data Bank (https://www.rcsb.org/, accessed on 19 July 2024). PyMOL 4.6.0 software was used to add hydrogen and remove water molecules from the receptor. Molecular docking of the 3D minimum-energy ligands and receptor was performed using the AutoDock vina tool 1.5.7.

## 3. Results and Discussion

### 3.1. Fast Determination of Small Peptides Based on LC–MS MS/MS with FBMN and PEAKS Studio

To rapidly determine and annotate small peptides, AAs, and other compounds in *L. reuteri*, FBMN analysis was employed. FBMN could generate clusters based on the similarity of the MS/MS ion fragments of the compounds, which could recommend the structure of unknown structures based on annotated nodes. Here, an FBMN with 1151 nodes and 98 clusters was processed. Fast annotation was first achieved based on databases in GNPS (MassBank and NIST, etc.), resulting in 18 annotated peptides, one AA derivative (lactoyl-Leu), and two metabolites (1-methyl-1,2,3,4-tetrahydro-β-carboline-3-carboxylic acid and 1,3-diphenylguanidine) (Figure S1, Table S1). Among the annotated peptides, not only line peptides but also some CDPs were identified. For example, as shown by the MS/MS mirror match of the node at *m*/*z* 261.124 (Figure S2A), the fragment ions at *m*/*z* 120.08 [M + H − C_6_H_7_NO_3_]^+^, 103.05 [M + H − C_6_H_10_N_2_O_3_]^+^, 91.05 [M + H − C_7_H_10_N_2_O_3_]^+^, 86.06 [M + H − C_10_H_9_NO_2_]^+^, and 68.05 [M + H − C_4_H_6_N]^+^ were matched with the MS/MS of cyclo(Phe-Hyp). Then, for the node at *m*/*z* 277.12, the fragment ions at *m*/*z* 231.11 [M + H − CO − H_2_O]^+^, 166.09 [M + H − C_5_H_5_NO_2_]^+^, 120.08 [M + H − C_6_H_7_NO_4_]^+^, and 84.04 [M + H − C_10_H_11_NO_3_]^+^ were matched to PyroGlu-Phe (Figure S2B). However, database matching relies on the coverage and quality of the MS/MS spectra in the database, which limits the annotation of compounds with little determinations or with new structures. As a result, in our research, only a few nodes were matched with the spectrum in the GNPS database, and there were still a large number of unannotated nodes in the molecular network (MN) (Figure S1). In this case, further identification of these nodes was required.

To dig more peptides, the samples were then analyzed by PEAKS studio based on a de novo sequencing strategy, which could annotate peptides by the sequential assignment of fragments [29]. Furthermore, a further enrichment of peptides was performed by MCX-SPE and these enriched samples were added to the de novo sequencing. With the help of de novo sequencing, a total of 133 peptides and three AAs, Val, lactoyl-Pro, and lactoyl-Phe, with screening scores above 80 were annotated and visualized on the MN (Figure S3, Table S2). For example, the fragment ions of the node at *m*/*z* 439.29 mainly consisted of the b and y ions of LPPL (Figure S4). The fragment ion at *m*/*z* 70.07 for [C_4_H_7_N]^+^ was generated by proline, at *m*/*z* 114.09 (b1), *m*/*z* 211.12 (b2), and *m*/*z* 308.20 (b3), as well as at *m*/*z* 132.10 (y1), *m*/*z* 229.15 (y2), and *m*/*z* 326.21 (y3), confirming the presence of a series of LPPL sequences. Among the de novo sequencing results, most of these peptides were dipeptides, tetrapeptides, pentapeptides, hexapeptides, heptapeptides, and decapeptides, while no cyclic peptides were identified.

Taken together, 151 compounds were rapidly identified using FBMN coupled with de novo sequencing, 122 of which were peptides. Among them, 3 peptides, cyclo(Tyr-Pro), Pro-Val, and Leu-Glu were commonly identified by both methods. Interestingly, the node at *m*/*z* 261.12 was annotated as cyclo(Tyr-Pro) by the FBMN database, while de novo sequencing recognized it as dehydrated Tyr-Pro. A further investigation of the MS/MS spectrum of this node was performed, according to the fragment ions at 155.08 [M + H − C_7_H_6_O]^+^, 136.08 [M + H − C_6_H_7_NO_2_]^+^, and 70.07 [M + H − C_10_H_9_NO_3_]^+^, which indicated the existence of a hexahydropyrrolo[1,2-a]pyrazine-1,4-dione structure and double amido bonds, and it was confirmed as cyclo(Tyr-Pro) (Figure S5). It was observed that de novo sequencing was challenging in identifying cyclic peptides. In addition, de novo could not distinguish between some isomers, such as leucine/isoleucine. This reminded us of the necessity for further analysis of these results, as well as the existence of numerous unknown nodes in the network clusters that were awaiting annotation. It was hypothesized that additional potential peptides might be identified in the clusters based on the characteristics of FBMN.

### 3.2. Thorough Profile of Peptides Based on FBMN

Due to the structural similarity of the different clusters, the cluster characteristics of the established FBMN were analyzed. The annotated peptides were mainly clustered in cluster-1, cluster-2, and cluster-3 (Figure S6). Among them, derived AAs and the majority of dipeptides were observed in cluster-1. Cluster-2 contained mainly aggregated CDPs and PyroGlu-containing dipeptides, while the tetrapeptides were mainly clustered in cluster-3. With the help of the clustering characteristics of structural analogs, unknown nodes could be quickly identified based on annotated nodes. Since the limitations of cyclic peptide identification mentioned above and the various bioactivities of CDPs, CDPs in MN were focused on for identification.

**Figure 1:**
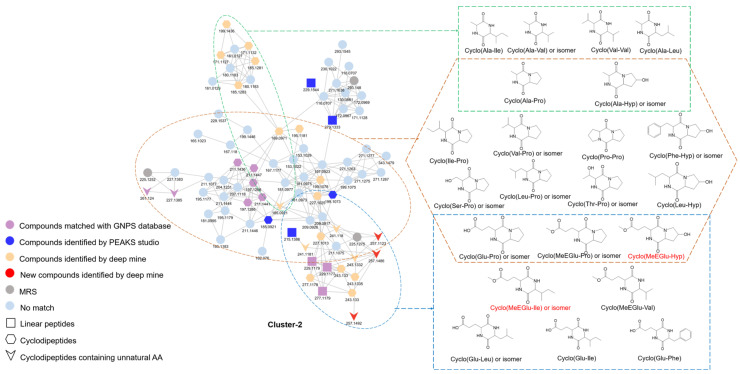
Proposed structures of the identified CDPs from cluster-2 of thorough profile by three methods in *L. reuteri*. CDPs containing Ala (green box), Pro and Hyp (orange box), and Glu and MeEGlu (blue box) are indicated.

Combined with the previously identified CDPs, a total of 53 CDPs including 15 unnatural AA-containing CDPs were annotated (Table 1), and most of the CDPs formed aggregates according to their AA composition (Figure S6). For instance, in cluster-2, the structurally similar CDPs were categorized into three groups, which were CDPs containing Ala (green box), Pro and Hyp (orange box), as well as Glu and MeEGlu (blue box) (Figure 1). Identification of unknown nodes could be carried out by grouping clusters, differences in precursor ions, and characteristic fragment ions. Such as nodes at *m*/*z* 197.13 and 211.14 in the orange box, which were annotated as cyclo(Val-Pro) and cyclo(Leu-Pro) or its isomer, respectively, were aggregated by the same specific fragment ions at *m*/*z* 70.07 and *m*/*z* 98.06 from the Pro residue. Additionally, a node at *m*/*z* 169.10 situated at the intersection of the orange box and the green box suggested a possible AA combination of Ala and Pro/Hyp. Then, according to the mass differences of 28 Da (-C_2_H_4_) and 42 Da (-C_3_H_6_) compared with the nodes of cyclo(Val-Pro) and cyclo(Leu-Pro) isomers, respectively, this node was annotated as cyclo(Ala-Pro) (Figure S7). Moreover, for the dehydrated dipeptides and CDPs which were hard to distinguish by de novo sequencing, clusters could help in their differentiation. Our finding indicated that nodes of dehydrated Pro-Ser and Thr-Pro identified by de novo sequencing were distributed in cluster-2, suggesting a high probability to be CDPs. The CDP characters observed in their MS spectra further proved this indication. Therefore, they were revised and annotated as CDPs, cyclo(Ser-Pro) and cyclo(Thr-Pro) (Figure 1 and Figure S8).

Specifically, four new peptides, cyclo(MeEGlu-Hyp), cyclo(MeEGlu-Ile) with its isomer, and cyclo(PyroGlu-Tyr), were found in *L. reuteri* for the first time based on these characteristics (Figure 1 and Table 1). The node at *m*/*z* 257.11 was in the common area of the yellow and blue boxes from cluster-2, which was associated with compounds containing Glu/Pro. The fragment ions at m/z 86.06 and 116.07 were characteristic immonium ions of Hyp and MeEGlu, respectively. In addition, the fragment of ions at *m*/*z* 239.10 [M + H − H_2_O]^+^, *m*/*z* 225.09 [M + H − CH_3_OH]^+^, and *m*/*z* 197.09 [M + H − CH_3_OH − CO]^+^ revealed the existing of methoxy group from the MeEGlu residue and the hydroxyl group Hyp residue. Therefore, this node was annotated as cyclo(MeEGlu-Hyp) (Figure S9A). Moreover, two nodes at *m*/*z* 257.1486 and 257.1492 in the yellow box generated the immonium ions of MeEGlu at *m*/*z* 116.07 and the loss of 32 Da (-CH_3_OH) (Figure S9B,C). Thus, it was hypothesized that they had the AA composition of MeEGlu. Owing to the presence of characteristic ions at *m*/*z* 86.10 and 69.07 from Ile, which were same as its nearby node at *m*/*z* 243.13 [cyclo(Glu-Ile)], indicating the composition of the Ile residue. Thus, these two nodes were identified as cyclo(MeEGlu-Ile) and its isomer (Figure S9B,C). Additionally, the node at *m*/*z* 275.10 in cluster-4 was 18 Da (H_2_O) less than the node at *m*/*z* 293.1119 of cyclo(Glu-Tyr) (Figure S10A). The fragment ion at *m*/*z* 136.08, was the same as the immonium ion of Tyr from cyclo(Glu-Tyr), indicating the composition of Tyr. Combined with the fragment ion at *m*/*z* 84.04, the immonium ion of PyroGlu, the node at *m*/*z* 275.10 was identified as cyclo(PyroGlu-Tyr) (Figure S10B).

In particular, the FBMN method enabled the preliminary and rapid discrimination of isomers. For structural isomers, the distinction could be made by cluster characteristics. For example, cyclo(Tyr-Hyp) and cyclo(Glu-Phe) have the same molecular composition as C_14_H_16_N_2_O_4_, while their nodes were separated into different clusters. For CDPs containing Leu/Ile, it is possible to use FBMN features along with characteristic ions to quickly distinguish them. For instance, compounds at *m*/*z* 185.1283 [cyclo(Ala-Leu/Ile)] displayed two nodes on the MN based on their different retention times (RTs). After reconfirming their characteristic ions, the node that eluted earlier was cyclo(Ala-Ile), and the other was cyclo(Ala-Leu) (Figure S11). Besides, since AAs (except Gly) have chiral carbons, CDPs could be stereoisomerized [30]. For this type of isomer, the differences in their RTs were used for their distinction on C18 reversed-phase (RP) chromatography [31]. According to the LC retention times, there were possibly 16 pairs of diastereoisomeric CDP isomers detected in this study.

**Figure 2:**
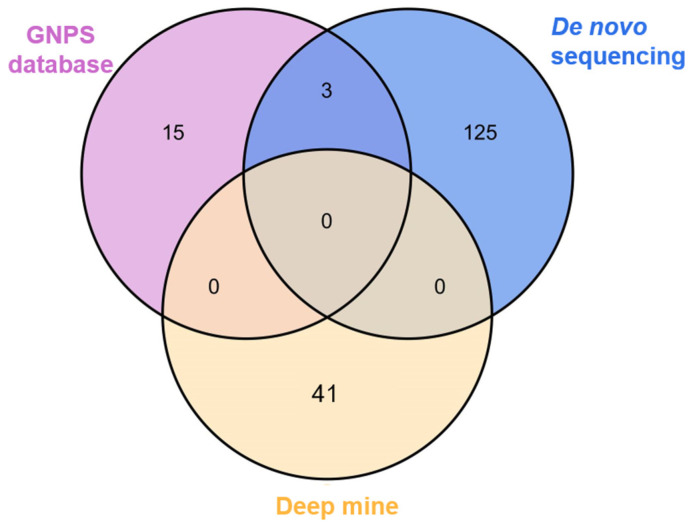
Peptides identified by GNPS database, de novo sequencing and deep mine.

Taken together, a total of 192 compounds, including 184 peptides, were rapidly identified by thorough analysis of FBMN. In Figure 2, the Venn diagram displays the complementarity between GNPS database matching, de novo sequencing by PEAKS, and deep mining approaches. The peptides detected by the GNPS database matching (purple section) and de novo sequencing (blue section) approaches were known peptides, and there were three duplicates of the identification results. The yellow section has no intersection with the other two methods, suggesting that FBMN-based deep mining could uncover more unknown peptides, including novel peptides. In addition, the number of CDPs identified by deep mining was higher than that of the GNPS database method, which improved efficiency of CDPs identification. Notably, with the interest of CDPs, 53 CDPs, including 15 unnatural AA containing CDPs with three types of unnatural AAs (Hyp, MeEGlu, and PyroGlu), in *L. reuteri* were determined. As previously described, CDPs as a kind of cyclic peptide with great bioavailability and potential beneficial activities were reported to have activities that might be influenced by their consisting AAs. Hyp-containing CDPs were found to possess beneficial activities [32,33], and unnatural AAs containing CDPs were also reported to be worthy of investigation [34]. Therefore, the CDPs we found by thorough analysis of the FBMN strategy might have potential for generating biological activities, which are worthy of further investigation.

### 3.3. Isolation and Identification of Novel Peptide

As new CDPs were identified, if the identification established through analysis of FBMN was confident, a compound of greatest interest was chosen to validate its structure. In this case, cyclo(MeEGlu-Hyp) (the node at *m*/*z* 257.1123) was initially isolated and identified by NMR after several chromatographic separation and purification steps.

**Figure 3:**
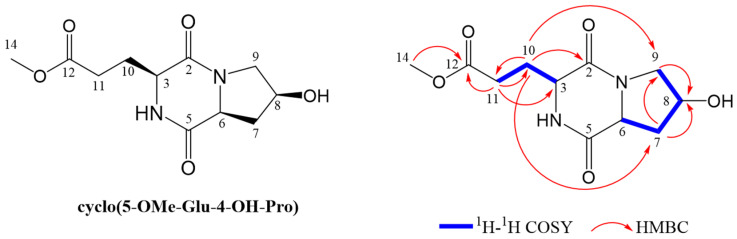
Structures of cyclo(5-OMe-Glu-4-OH-Pro) and its key ^1^H–^1^H COSY and HMBC correlations.

Cyclo(MeEGlu-Hyp) was first obtained as an amorphous transparent solid and identified as a novel unnatural AA-containing CDP. The molecular formula was assigned as C_11_H_16_N_2_O_5_ based on the ion peak at *m*/*z* 257.1139 ([M + H]^+^, calcd for 257.1132) by high-resolution ESI-MS analysis, indicating five degrees of unsaturation. As shown in Table S3, Figure 3 and Figures S12–S18, the bicyclic structure of 2,5-diketopiperazine (DKP) could be inferred from the ^1^H and ^13^C chemical shifts, coupling constants (*J*), ^1^H–^1^H COSY interpretations, HSQC signal assignments, and HMBC associations [35,36]. The hydroxyproline moiety of pyrrolidinol was observed according to an *N*-substituted methyne group at *δ* 4.50 (1H, ddd, *J* = 11.1, 6.2, 1.2 Hz), an *O*-substituted methyne group at *δ* 4.47 (1H, t, *J* = 4.3 Hz), and two methylene groups at *δ* 2.10 (1H, ddd, *J* = 13.0, 11.4, 4.3 Hz)/2.29 (1H, dd, *J* = 6.2, 13.2 Hz) and *δ* 3.43 (1H, d, *J* = 12.6 Hz)/3.72 (1H, dd, *J* = 13.0, 4.9 Hz) (Figures S12–S17). Sequential ^1^H–^1^H COSY interpretations (Figure S15) among H-6 (*δ* 4.50), H-7 (*δ* 2.10 and 2.29), H-8 (*δ* 4.47), and H-9 (*δ* 3.43 and 3.72), as well as the HMBC correlations from H-7 to *δ*_C_ 69.8 (C-8) and *δ*_C_ 56.0 (C-9), and from H-9 to C-8 (Figure S17), suggested their connection sequence and further illustrated that the 4-position of the pyrrolidinol moiety was substituted with a hydroxyl group. In addition, the carbon signals of C-2 (*δ* 168.6) and C-5 (*δ* 173.8) from the ^13^C NMR and DEPT spectra indicated the existence of two amide carbon groups. Two methylene signals of the 5-methyl hydrogen glutamate at *δ*_H-11_ 2.50 (1H, d, *J* = 2.50 Hz)/2.51 (1H, d, *J* = 3.10 Hz) and a methyl signal at *δ*_H-14_ 3.67 (3H, s) were correlated with the quaternary carbon C-12 (*δ* 176.2) via HMBC, suggesting the presence of a methoxy group. Furthermore, from the NOESY spectrum data, H-3, H-6, and H-8 were interrelated with each other, demonstrating that the three protons were on the same side (Figure S18). UHPLC trace of extracted ion chromatogram (EIC) spectra of *m*/*z* = 257.1132 in the collected fraction and purified sample were used to confirm that the isolation identification was the same as the FBMN identification (Figure S19). In this case, cyclo(MeEGlu-Hyp) was identified as cyclo(5-OMe-Glu-4-OH-Pro).

### 3.4. Prediction of the Potential Anti-Inflammatory Activity of Peptides by Molecular Docking

Many peptides have been reported to possess biological activities. Among several detected CDPs, there might be some potentially bioactive peptides contributing to the anti-inflammatory activity of *L. reuteri*. Therefore, a preliminary prediction of their anti-inflammatory activity was performed using computer simulations. PeptideRanker is a valuable tool in the analysis of bioactive peptides and has proved to aid in the discovery of peptides with potential therapeutic applications [37,38,39]. AIPpred is a sequence-based prediction tool for identifying anti-inflammatory peptides (AIPs) [40]. An initial screening of chain peptides with anti-inflammatory activity was carried out by PeptideRanker with AIPpred before molecular docking verification. The 19 peptides based on sequence with PeptideRanker prediction scores ≥ 0.5 and with AIPpred prediction scores ≥ 0.342 were screened for further molecular docking (Table S4). Molecular docking is an effective technique for simulating the interactions between ligands and receptors. Several anti-inflammatory activity-related protein targets that have gained much attention recently were selected, including NEK7, Cat C, GSDMD, and the TLR4/MD2 complex. Additionally, some classic anti-inflammatory targets, such as TNF-α, IL-6, and IL-1β, were selected to evaluate the potential anti-inflammatory activity of the identified peptides.

We examined the docking affinity binding energies of the identified peptides for the seven target proteins mentioned above (Table S5). It was observed that CDPs have better binding abilities to NEK7, Cat C, and GSDMD compared to the anti-inflammatory drug Rolipram [41,42]. As shown in Table 2, cyclo(Glu-Phe) interacted with NEK7 and had a binding energy of −7.6 kcal/mol (Rolipram: −7.2 kcal/mol). This indicated that some CDPs might bind to NEK7 with the potential to block the NEK7-NLRP3 interaction, thereby preventing NLRP3 activation and ameliorating inflammatory diseases [43]. Cat C was reported to be an attractive target for the treatment of neutrophil serine protease (NSP)-related inflammatory diseases [44]. Cyclo(Phe-Hyp) had a binding energy of −8.0 kcal/mol with Cat C (Rolipram: −7.7 kcal/mol). This suggested that some CDPs probably associate with Cat C, thereby inhibiting target protein activity and downstream NSPs activation. Additionally, cyclo(PyroGlu-Tyr) had a binding energy of −7.1 kcal/mol to GSDMD (Rolipram: −6.3 kcal/mol). GSDMD is a Gasdermin family protein, that is the key executor of pyroptotic cell death and is thought to be a novel therapeutic target for inflammatory diseases [45]. This indicated that it might interact with GSDMD as an inhibitor to inhibit its activation and the release of pro-inflammatory factors [46].

**Figure 4:**
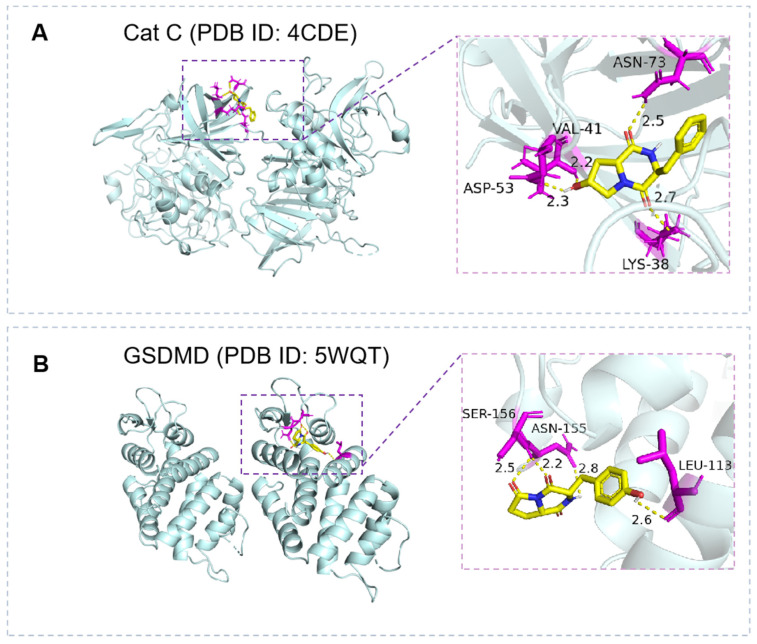
Molecular docking results of (**A**) cyclo(Phe-Hyp) with Cat C (PDB ID: 4CDE) and (**B**) cyclo(PyroGlu-Tyr) with GSDMD (PDB ID: 5WQT).

Based on these results, CDPs containing unnatural AAs showed better binding abilities. We hypothesise unnatural AAs might benefit CDPs’ activities. It could be noted that, in 3D diagrams of cyclo(Phe-Hyp) with Cat C (PDB ID: 4CDE) and cyclo(PyroGlu-Tyr) with GSDMD (PDB ID: 5WQT) (Figure 4), both PyroGlu and Hyp residues showed contribution to the protein bindings. Based on the report, the peptide fraction from sturgeon muscle hydrolyzed by pepsin with a high content of Tyr was reported to show stronger anti-inflammatory activity than peptides containing other AAs [47]. Additionally, CDPs containing Hyp have been reported to have good anti-inflammatory effects [32,48]. Moreover, the peptides identified in this work, cyclo(Leu-Hyp), cyclo(Ile-Pro), cyclo(Leu-Pro), and cyclo(Phe-Hyp), have been reported to show good inhibitory effects on TNF-α and IL-6 in lipopolysaccharide-stimulated macrophages [49]. Furthermore, cyclo(Gly-Pro) detected in this study was also discovered to attenuate nociceptive behavior and the inflammatory response in mice [50]. Thus, we considered that the existence of unnatural AAs in peptides might contribute to their anti-inflammatory activity.

## 4. Conclusions

In this study, we performed a method incorporating FBMN and de novo sequencing to deep mine the peptides, AAs, and other compounds in *L. reuteri* based on LC–MS. A total of 192 compounds, including 184 peptides, 6 AAs or AA derivatives, and 2 other compounds, were identified rapidly. Notably, 53 CDPs were annotated, including 15 unnatural AA-containing CDPs and 4 novel CDPs. Subsequently, the isolation and structural characterization of the novel compound was conducted, which were in accordance with the identification results. Moreover, molecular docking was used to predict the potential anti-inflammatory activity of the identified peptides. In particular, CDPs containing unnatural AAs exhibited better interactions with the anti-inflammatory-related target proteins, including NEK7, Cat C, GSDMD, etc. It was considered that the existence of unnatural AAs in CDPs might contribute to their anti-inflammatory activity. These results provide a valuable strategy for the rapid identification of peptides including CDPs. Additionally, it also reveals the substance basis for the potential anti-inflammatory effects exerted by *L. reuteri*.

## Figures and Tables

**Table 1:** The CDPs generated in *L. reuteri*.

No.	Compound Name	Molecular Formula	*m*/*z*	RT (min)
1	cyclo(Gly-Pro)	C_7_H_10_N_2_O_2_	155.0817	3.62
2	cyclo(Ala-Pro)	C_8_H_12_N_2_O_2_	169.0971	5.05
3	cyclo(Ala-Val) or isomer	C_8_H_14_N_2_O_2_	171.1127	7.57
4	cyclo(Ala-Val) or isomer	C_8_H_14_N_2_O_2_	171.1132	9.68
5	cyclo(Ser-Pro) or isomer	C_8_H_12_N_2_O_3_	185.0918	1.20
6	cyclo(Ser-Pro) or isomer	C_8_H_12_N_2_O_3_	185.0921	2.85
7	cyclo(Ala-Hyp) or isomer	C_8_H_12_N_2_O_3_	185.0921	2.20
8	cyclo(Ala-Hyp) or isomer	C_8_H_12_N_2_O_3_	185.0921	2.52
9	cyclo(Ala-Leu)	C_9_H_16_N_2_O_2_	185.1281	13.34
10	cyclo(Ala-Ile)	C_9_H_16_N_2_O_2_	185.1283	12.02
11	cyclo(Pro-Pro)	C_10_H_14_N_2_O_2_	195.1181	10.03
12	cyclo(Val-Pro) or isomer	C_10_H_16_N_2_O_2_	197.1286	12.71
13	cyclo(Val-Pro) or isomer	C_10_H_16_N_2_O_2_	197.1286	13.11
14	cyclo(Thr-Pro) or isomer	C_9_H_14_N_2_O_3_	199.1073	3.15
15	cyclo(Thr-Pro) or isomer	C_9_H_14_N_2_O_3_	199.1078	4.21
16	cyclo(Val-Val)	C_10_H_18_N_2_O_2_	199.1436	18.61
17	cyclo(Ser-Leu)	C_9_H_16_N_2_O_3_	201.1222	6.32
18	cyclo(Leu-Pro) or isomer	C_11_H_18_N_2_O_2_	211.1436	18.79
19	cyclo(Ile-Pro)	C_11_H_18_N_2_O_2_	211.1441	17.99
20	cyclo(Leu-Pro) or isomer	C_11_H1_8_N_2_O_2_	211.1447	19.16
21	cyclo(Asn-Pro)	C_9_H_13_N_3_O_3_	212.1040	2.24
22	cyclo(Val-Leu)	C_11_H_20_N_2_O_2_	213.1594	23.37
23	cyclo(Asn-Val)	C_9_H_15_N_3_O_3_	214.1181	2.90
24	cyclo(Asp-Val) or isomer	C_9_H_14_N_2_O_4_	215.1020	7.26
25	cyclo(Asp-Val) or isomer	C_9_H_14_N_2_O_4_	215.1030	9.41
26	cyclo(Glu-Pro) or isomer	C_10_H_14_N_2_O_4_	227.1013	6.47
27	cyclo(Glu-Pro) or isomer	C_10_H_14_N_2_O_4_	227.1025	7.64
28	cyclo(Leu-Hyp) or isomer	C_11_H_18_N_2_O_3_	227.1385	14.50
29	cyclo(Leu-Hyp) or isomer	C_11_H_18_N_2_O_3_	227.1387	15.15
30	cyclo(Asn-Ile)	C_10_H_17_N_3_O_3_	228.1343	6.09
31	cyclo(Asn-Leu)	C_10_H_17_N_3_O_3_	228.1343	6.70
32	cyclo(Asp-Ile) or isomer	C_10_H_16_N_2_O_4_	229.1171	12.13
33	cyclo(Asp-Ile) or isomer	C_10_H_16_N_2_O_4_	229.1174	11.53
34	cyclo(MeEGlu-Pro) or isomer	C_11_H_16_N_2_O_4_	241.1180	11.88
35	cyclo(MeEGlu-Pro) or isomer	C_11_H_16_N_2_O_4_	241.1181	13.12
36	cyclo(Glu-Leu) or isomer	C_11_H_18_N_2_O_4_	243.1330	17.17
37	cyclo(Glu-Leu) or isomer	C_11_H_18_N_2_O_4_	243.1330	18.51
38	cyclo(MeEGlu-Val)	C_11_H_18_N_2_O_4_	243.1332	16.45
39	cyclo(Glu-Ile)	C_11_H_18_N_2_O_4_	243.1335	14.83
40	cyclo(Phe-Pro)	C_14_H_16_N_2_O_2_	245.1280	22.23
41	cyclo(MeEGlu-Hyp) *	C_11_H_16_N_2_O_3_	257.1123	8.80
42	cyclo(MeEGlu-Ile) or isomer *	C_12_H_20_N_2_O_4_	257.1486	23.26
43	cyclo(MeEGlu-Ile) or isomer *	C_12_H_20_N_2_O_4_	257.1492	20.82
44	cyclo(Phe-Hyp) or isomer	C_11_H_18_N_2_O_3_	261.1230	16.84
45	cyclo(Tyr-Pro)	C_14_H_16_N_2_O_3_	261.1237	13.31
46	cyclo(Phe-Hyp) or isomer	C_11_H_18_N_2_O_3_	261.1240	17.80
47	cyclo(PyroGlu-Tyr) *	C_14_H_14_N_2_O_4_	275.1037	11.45
48	cyclo(Tyr-Hyp) or isomer	C_14_H_16_N_2_O_4_	277.1171	12.43
49	cyclo(Tyr-Hyp) or isomer	C_14_H_16_N_2_O_4_	277.1178	12.10
50	cyclo(Glu-Phe)	C_14_H_16_N_2_O_4_	277.1178	20.30
51	cyclo(Tyr-Asp)	C_13_H_14_N_2_O_5_	279.1332	10.06
52	cyclo(Glu-Tyr) or isomer	C_14_H_16_N_2_O_5_	293.1119	10.26
53	cyclo(Glu-Tyr) or isomer	C_14_H_16_N_2_O_5_	293.1122	11.43

* Novel compound.

**Table 2:** Docking score of the partial peptides (docking score with NEK7 ≤ −6.0 kcal/mol) and the anti-inflammatory drug Rolipram with NEK7 (PDB ID: 2WQN), Cat C (PDB ID: 4CDE), and GSDMD (PDB ID: 5WQT).

		Binding Energy (kcal/mol)		
No.	Ligand	NEK7	Cat C	GSDMD
1	cyclo(Glu-Phe)	−7.6	−7.6	−6.2
2	cyclo(Phe-Pro)	−7.5	−7.6	−6.6
3	cyclo(Tyr-Hyp)	−7.4	−7.6	−6.8
4	cyclo(Phe-Hyp)	−7.3	−8.0	−6.5
5	PyroGlu-Phe	−7.2	−7.8	−6.4
6	Rolipram	−7.2	−7.7	−6.3
7	cyclo(Tyr-Asp)	−7.1	−7.7	−6.6
8	cyclo(PyroGlu-Tyr)	−7.0	−7.4	−7.1
9	cyclo(Tyr-Pro)	−7.0	−7.2	−6.6
10	LPNLP	−7.0	−6.5	−6.5
11	VYPFPGPLPQ	−7.0	−7.8	−6.3
12	YPFELP	−7.0	−7.1	−6.7
13	cyclo(Glu-Tyr)	−6.9	−7.6	−6.4
14	WS(+14.02)	−6.8	−6.8	−5.9
15	PLLLP	−6.8	−6.5	−7.1
16	RMPPSP	−6.8	−6.9	−6.0
17	VYPFPGPLPE	−6.7	−6.8	−6.2
18	VYPFPGPLPN	−6.7	−6.7	−7.1
19	cyclo(MeEGlu-Hyp)	−6.6	−6.8	−5.6
20	cyclo(Glu-Leu)	−6.6	−6.5	−5.8
21	YPFPALP	−6.6	−7.7	−6.4
22	cyclo(Glu-Pro)	−6.5	−6.6	−5.6
23	L(+72.02)A	−6.5	−6.2	−5.0
24	LPLLP	−6.5	−7.3	−6.8
25	cyclo(Glu-Ile)	−6.4	−7.1	−5.3
26	cyclo(Val-Leu)	−6.4	−6.6	−5.9
27	cyclo(Leu-Pro)	−6.4	−6.4	−5.5
28	LPPL	−6.4	−6.3	−6.8
29	cyclo(Asn-Pro)	−6.4	−6.5	−5.4
30	VYPFPGPLEP	−6.4	−7.1	−6.3
31	cyclo(Leu-Hyp)	−6.3	−6.6	−5.8
32	cyclo(MeEGlu-Pro)	−6.3	−6.4	−5.9
33	cyclo(Val-Val)	−6.3	−6.3	−5.5
34	VAPFPEVFA	−6.3	−6.6	−7.7
35	YVPL	−6.2	−7.5	−6.7
36	cyclo(MeGlu-Ile)	−6.2	−6.6	−5.4
37	PLEFP	−6.2	−7.6	−6.5
38	TLEQLFPPVLVPVPNTPLP	−6.2	−6.4	−6.0
39	YPVEPF	−6.2	−7.7	−7.1
40	D(−18.01)APL	−6.1	−7.0	−5.9
41	cyclo(Asp-Val)	−6.1	−6.4	−5.5
42	cyclo(Ile-Pro)	−6.0	−6.9	−5.6
43	cyclo(MeEGlu-Val)	−6.0	−6.4	−5.9
44	P(+27.99)VSY	−6.0	−6.2	−6.0
45	E(+14.02)L	−6.0	−5.9	−4.9
46	cyclo(Asn-Val)	−6.0	−6.5	−5.7
47	cyclo(Asn-Leu)	−6.0	−6.6	−5.8
48	LPLPL	−6.0	−6.1	−6.0
49	YVPFPGPLEP	−6.0	−7.4	−7.0

## Data Availability

The data presented in this study are available on request from the corresponding author due to privacy.

## Supplementary Materials

The following supporting information can be downloaded at: https://www.mdpi.com/article/10.3390/metabo14090467/s1, Figures S1–S11: FBMN analysis data and MS/MS spectra of identified compounds in *L. reuteri*; Figures S12–S19: ^1^H, ^13^C, 2D NMR, and EIC spectra of cyclo(5-OMe-Glu-4-OH-Pro); Table S1: The compounds identified via FBMN; Table S2: The compounds identified by PEAKS studio; Table S3: ^1^H NMR (600 MHz, CD_3_OD), ^13^C NMR (150 MHz, CD_3_OD) HMBC, ^1^H–^1^H COSY and NOESY spectra data of cyclo(5-OMe-Glu-4-OH-Pro); Table S4: Sequences of the peptides with their scores generated by AIPpred (scores ≥ 0.342) and PeptideRanker (scores ≥ 0.5); Table S5: Docking score of peptides and Rolipram with NEK7 (PDB ID: 2WQN), Cat C (PDB ID: 4CDE), and GSDMD (PDB ID: 5WQT), the TLR4/MD2 complex (PDB ID: 3FXI), TNF-α (PDB ID: 1TNF), IL-6 (PDB ID: 1N26), and IL-1β (PDB ID: 3O4O).
