# Supplementary material for: A Deep Mining Strategy for Peptide Rapid Identification in Lactobacillus reuteri Based on LC–MS/MS Integrated with FBMN and De Novo Sequencing
Source: Metabolites. 2024 Aug 23;14(9):467. doi: 10.3390/metabo14090467 (PMC11434120; doi:10.3390/metabo14090467)
Supplement: Supplementary file 1 [file metabolites-14-00467-s001.zip › metabolites-3167841-supplementary.pdf]

Supporting information for

A Deep Mining Strategy for Peptide Rapid Identification in *Lactobacillus reuteri* Based on LC–MS/MS Integrated with FBMN and De Novo Sequencing

Yilang Zuo,[a] Shilin Gong,[a] Li Zhang,[a] Jie Zhou,[a] Jian-Lin Wu,[a,*] and Na Li[a,*]

State Key Laboratory of Quality Research in Chinese Medicine, Macau Institute for Applied Research in Medicine and Health, Macau University of Science and Technology, Taipa, Macau SAR 999078 , China; 2009853vct20002@student.must.edu.mo (Y.Z.); 21098532ct30001@student.must.edu.mo (S.G.); 2109853qct30001@student.must.edu.mo (L.Z.); 2009853zct20001@student.must.edu.mo (J.Z.)

* Correspondence: jlwu@must.edu.mo (J.-L.W.); nli@must.edu.mo (N.L.)

Figure Captions

Figure S1. FBMN analysis of compounds matched with GNPS database from *L. reuteri* sample.3
Figure S2. MS/MS spectra of nodes at *m/z* 261.12 [cyclo(Phe-Hyp)] (A) and 277.12 (PyroGlu-Phe) (B) and their structures, the black spectra in the upper represent the actual, and the green in the lower represent the spectra in the database. ······ 3
Figure S3. FBMN analysis combined with PEAKS studio of compounds in *L. reuteri*. ······ 4
Figure S4. MS/MS spectrum of the node at *m/z* 439.29 which identified as LPPL. ······ 4
Figure S5. MS/MS spectrum of node at *m/z* 261.1237 [cyclo(Tyr-Pro)] and its structure. ······ 4
Figure S6. Thorough analysis of FBMN and compounds annotation in *L. reuteri*. ······ 5
Figure S7. Anssociation of nodes at *m/z* 169.0917 with the annotated nodes at *m/z* 211.1436, 211.1447, and 197.1286 in cluster-2. ······ 5
Figure S8. MS/MS spectra of node at *m/z* 185.0921 with RT = 2.85 min [cyclo(Ser-Pro)] and 199.1073 with RT = 3.15 min [cyclo(Thr-Pro)] and their structures. ······ 5
Figure S9. MS/MS spectra of node at *m/z* 257.1123 [cyclo(MeEGlu-Hyp)], 257.1492 [cyclo(MeEGlu-Ile) or isomer], and 257.1486 [cyclo(MeEGlu-Ile) or isomer)], and their structures. ······ 6
Figure S10. (A) Anssociation of node at *m/z* 275.1037 with the annotated node at *m/z* 293.1119 in cluster-2. (B) MS/MS spectrum of node at *m/z* 275.1037 [cyclo(PyroGlu-Tyr)] and its structure. ······ 6
Figure S11. MS/MS spectra of node at *m/z* 185.1281 [cyclo(Ala-Leu)] and 185.1283 [cyclo(Ala-Ile)] and their structures. ······ 6
Figure S12. ^{1}H NMR spectrum of cyclo(5-OMe-Glu-4-OH-Pro). ······ 7
Figure S13. ^{13}C NMR spectrum of cyclo(5-OMe-Glu-4-OH-Pro). ······ 8
Figure S14. DEPT 135 NMR spectrum of cyclo(5-OMe-Glu-4-OH-Pro). ······ 9
Figure S15. ^{1}H-^{1}H COSY NMR spectrum of cyclo(5-OMe-Glu-4-OH-Pro). ······ 10
Figure S16. HSQC NMR spectrum of cyclo(5-OMe-Glu-4-OH-Pro). ······ 11
Figure S17. HMBC NMR spectrum of cyclo(5-OMe-Glu-4-OH-Pro). ······ 12
Figure S18. NOESY NMR spectrum of cyclo(5-OMe-Glu-4-OH-Pro). ······ 13
Figure S19. EIC spectra of cyclo(5-OMe-Glu-4-OH-Pro) (*m/z* = 257.1132) in collected fraction (A) and purified sample (B). ······ 13

Table Captions

Table S1. The compounds identified via FBMN. Compound name, *m/z*, and PI/Database found by FBMN. ······ 14
Table S2. The compounds identified by PEAKS studio. Compound name, *m/z*, and Type of modification found by PEAKS studio. ······ 14
Table S3. ^{1}H NMR (600 MHz, CD_3OD), ^{13}C NMR (150 MHz, CD_3OD) HMBC, ^{1}H-^{1}H COSY and NOESY spectra data of cyclo(5-OMe-Glu-4-OH-Pro). ······ 18
Table S4. Sequences of the peptides with their scores generated by AIPpred (scores ≥ 0.342) and PeptideRanker (scores ≥ 0.5). ······ 18
Table S5. Docking score of peptides and rolipram with NEK7 (PDB ID: 2WQN), Cat C (PDB ID: 4CDE), and GSDMD (PDB ID: 5WQT), the TLR4/MD2 complex (PDB ID: 3FXI), TNF-α (PDB ID: 1TNF), IL-6 (PDB ID: 1N26), and IL-1β (PDB ID: 3O4O). ······ 19

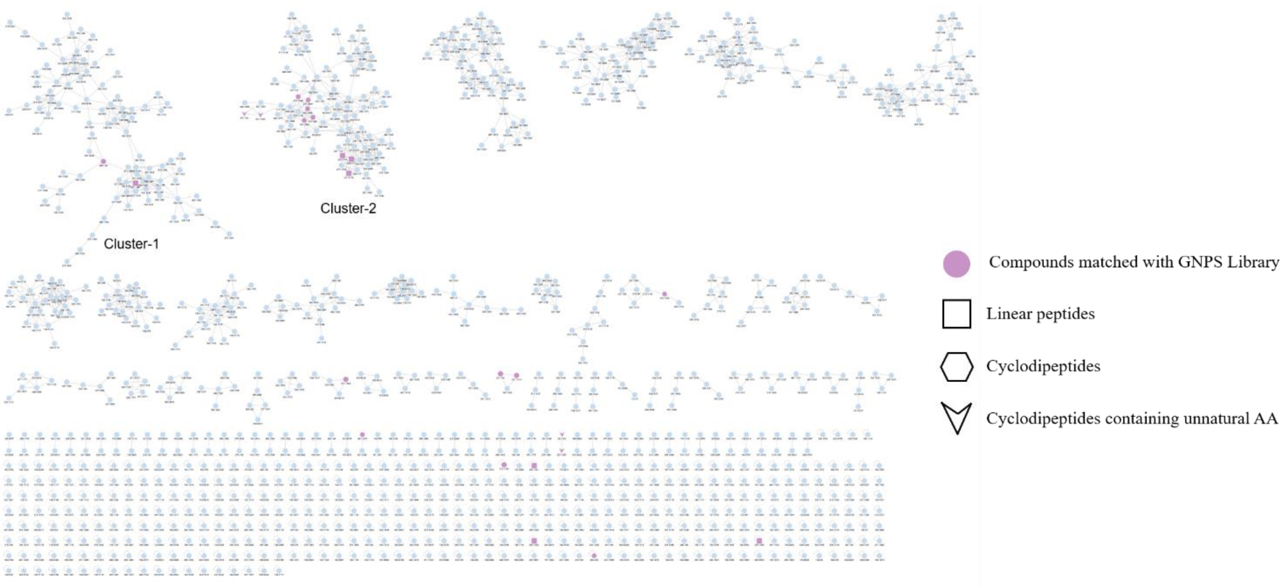

Figure S1. FBMN analysis of compounds matched with GNPS database from *L. reuteri* sample.

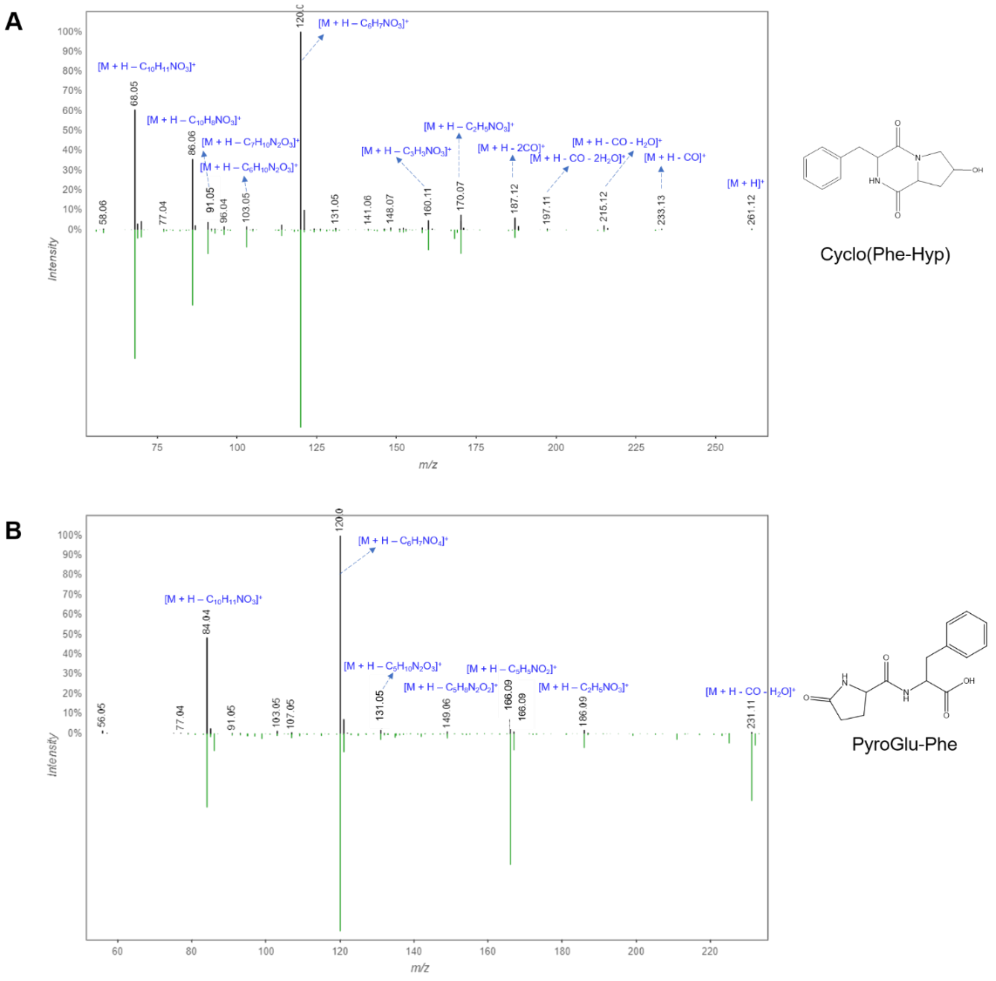

Figure S2. MS/MS spectra of nodes at *m/z* 261.12 [cyclo(Phe-Hyp)] (A) and 277.12 (PyroGlu-Phe) (B) and their structures, the black spectra in the upper represent the actual, and the green in the lower represent the spectra in the

database.

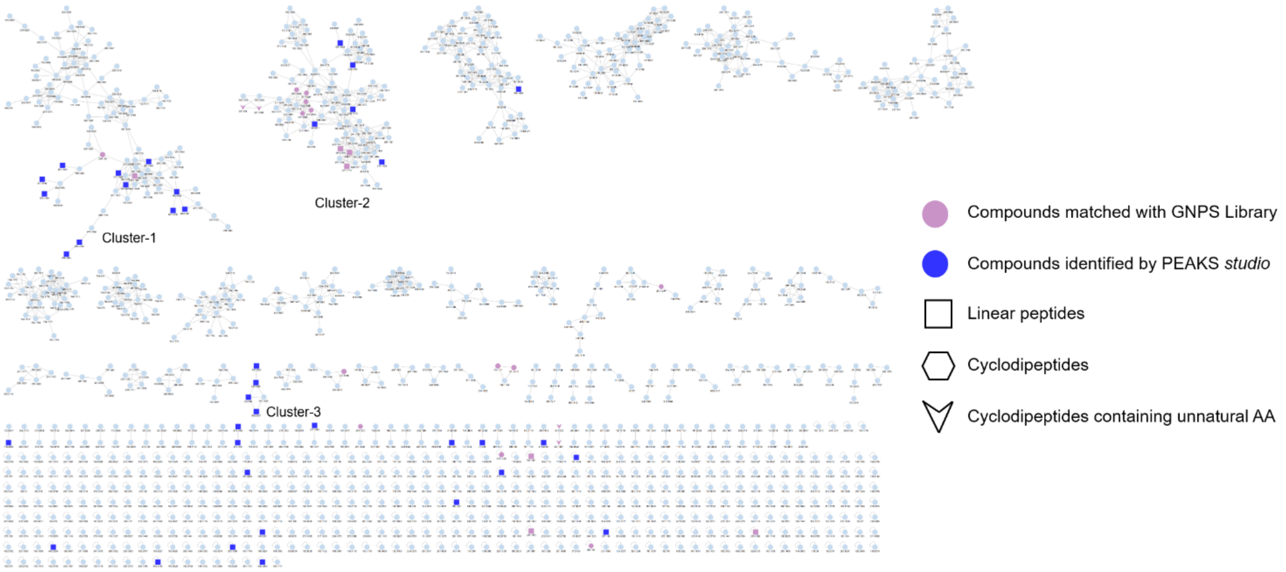

Figure S3. FBMN analysis combined with PEAKS studio of compounds in *L. reuteri*.

LPPL

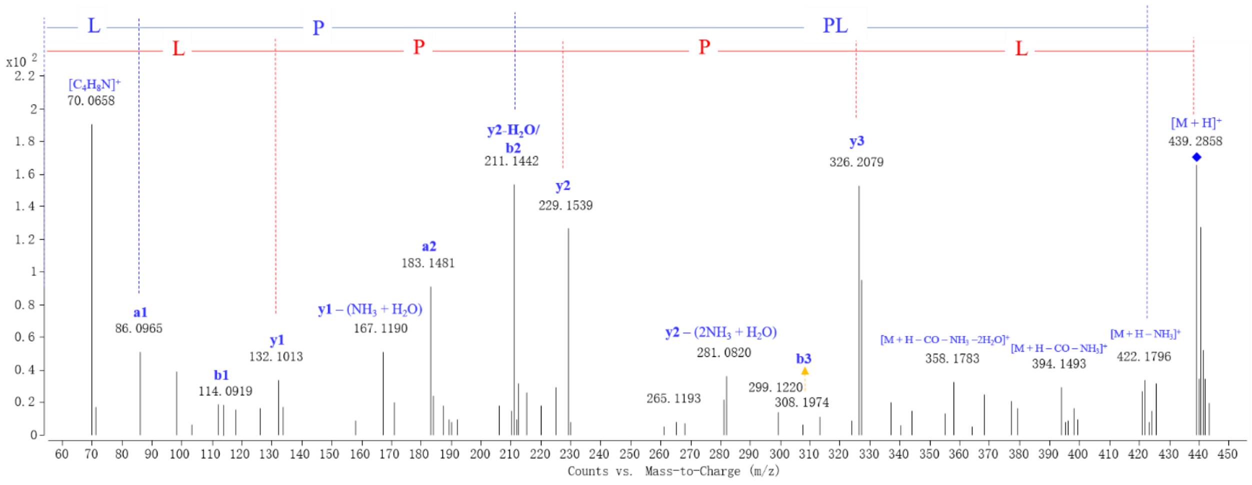

Figure S4. MS/MS spectrum of the node at *m/z* 439.29 which identified as LPPL.

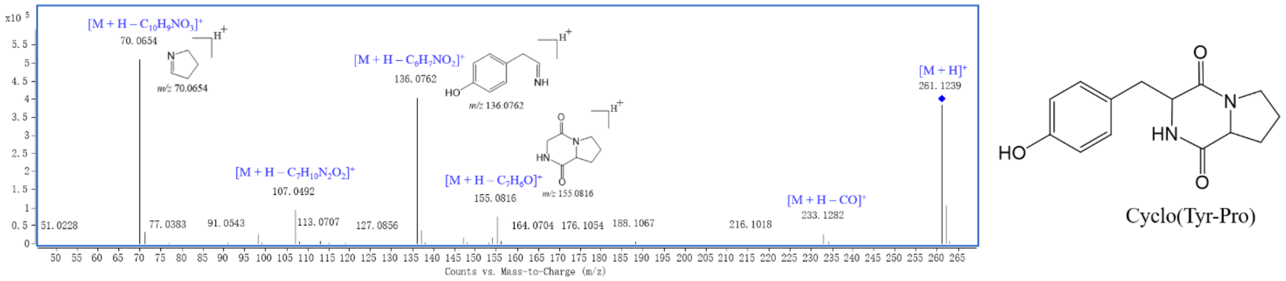

Figure S5. MS/MS spectrum of node at *m/z* 261.1237 [cyclo(Tyr-Pro)] and its structure.

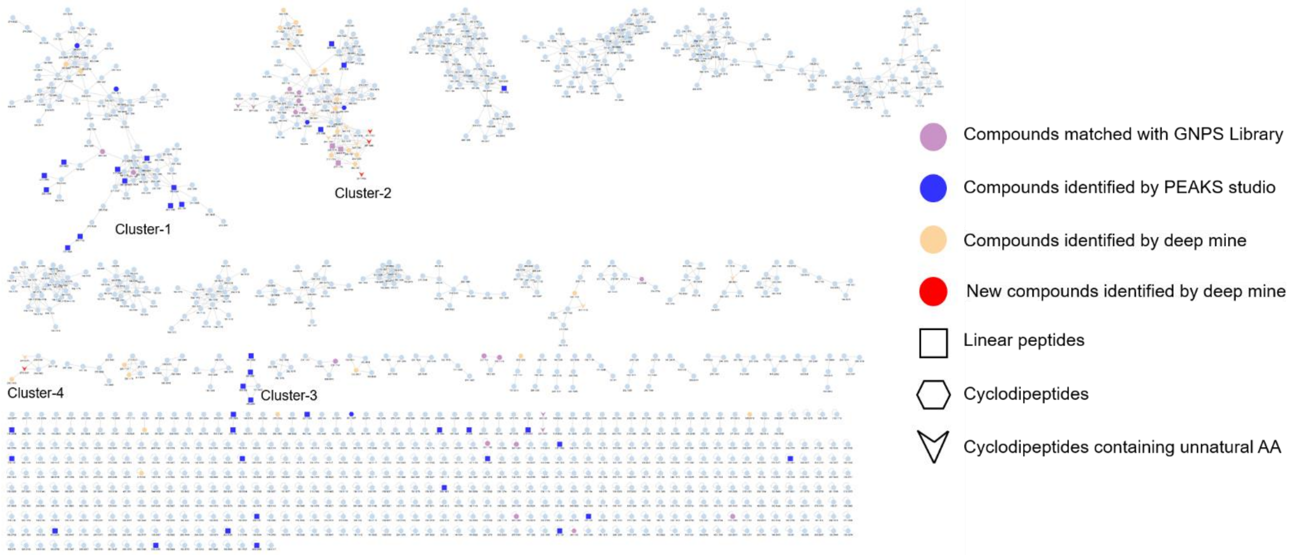

Figure S6. Thorough analysis of FBMN and compound annotation in *L. reuteri.*

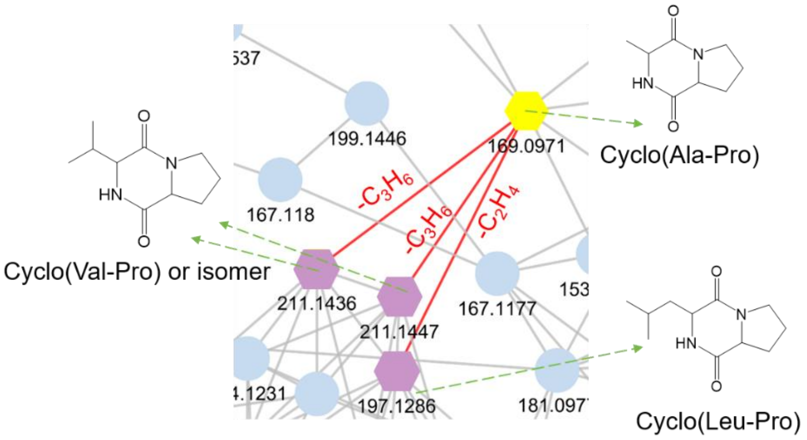

Figure S7. Anssociation of nodes at *m/z* 169.0917 with the annotated nodes at *m/z* 211.1436, 211.1447, and 197.1286 in the cluster-2.

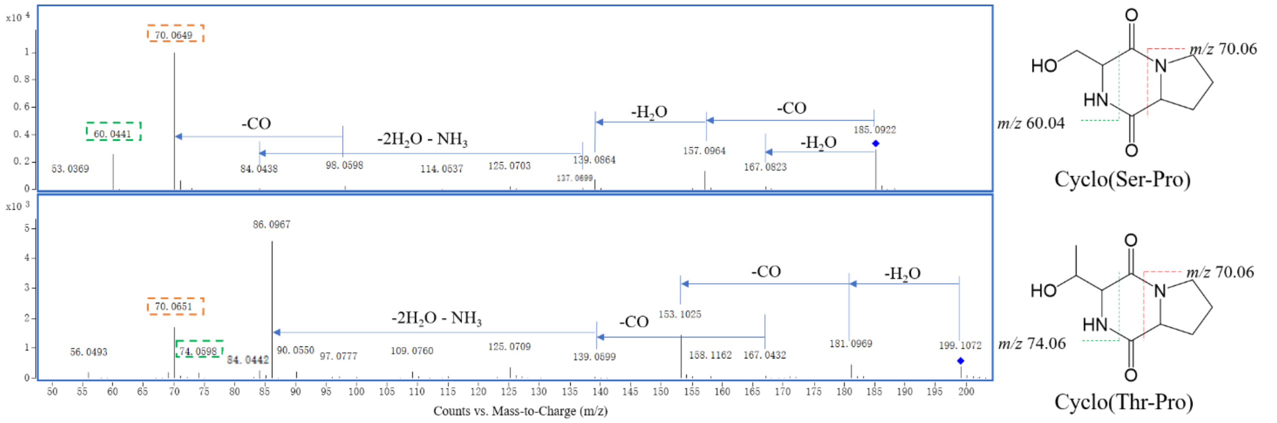

Figure S8. MS/MS spectra of node at *m/z* 185.0921 with RT = 2.85 min [cyclo(Ser-Pro)] and 199.1073 with RT = 3.15 min [cyclo(Thr-Pro)] and their structures.

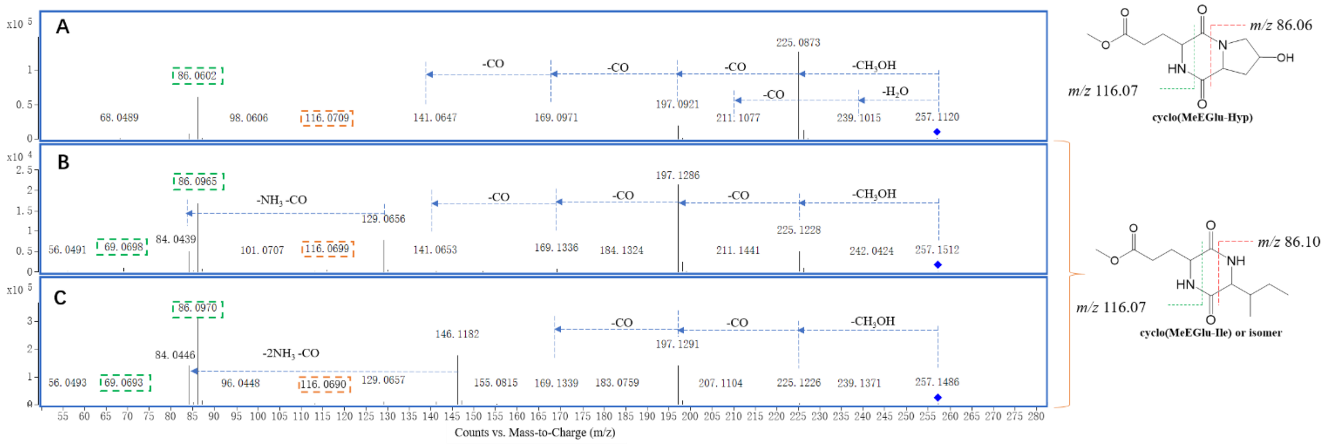

Figure S9. MS/MS spectra of node at *m/z* 257.1123 [cyclo(MeEGlu-Hyp)], 257.1492 [cyclo(MeEGlu-Ile) or isomer], and 257.1486 [cyclo(MeEGlu-Ile) or isomer)], and their structures.

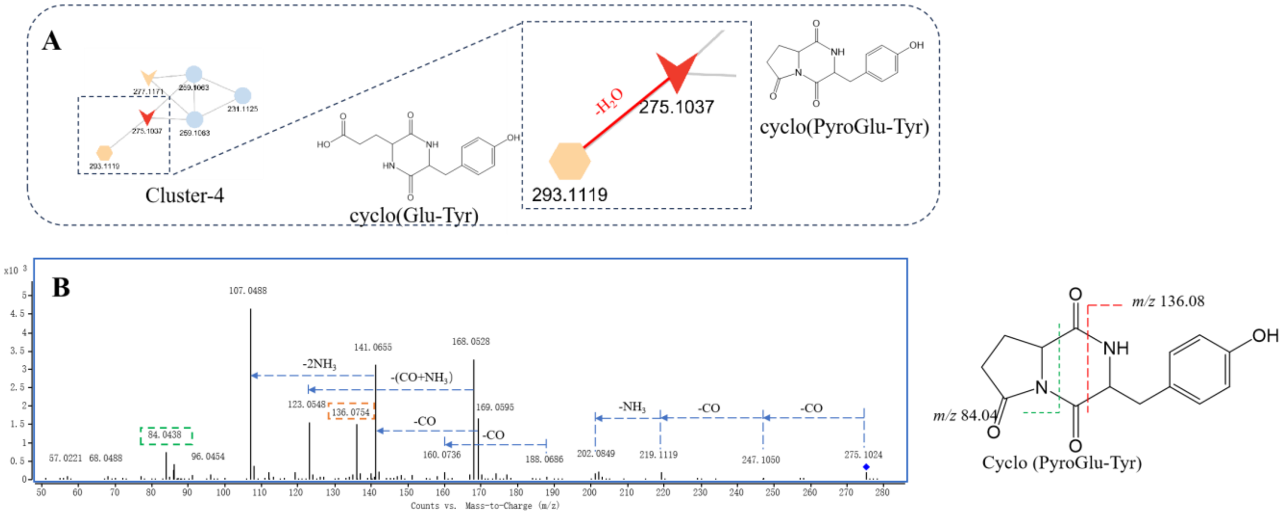

Figure S10. (A) Anssociation of node at *m/z* 275.1037 with the annotated node at *m/z* 293.1119 in cluster-2. (B) MS/MS spectrum of node at *m/z* 275.1037 [cyclo(PyroGlu-Tyr)] and its structure.

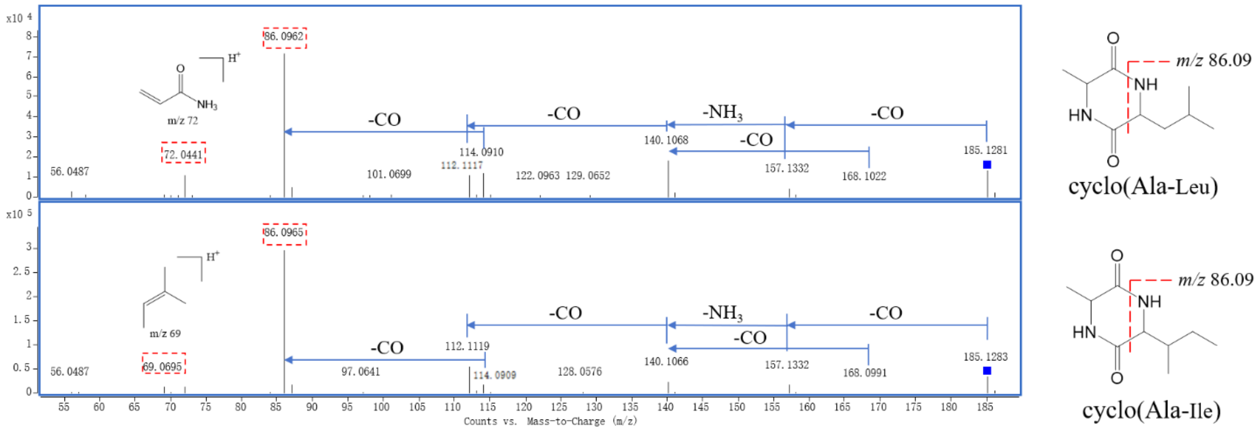

Figure S11. MS/MS spectra of node at *m/z* 185.1281 [cyclo(Ala-Leu)] and 185.1283 [cyclo(Ala-Ile)] and their

structures.

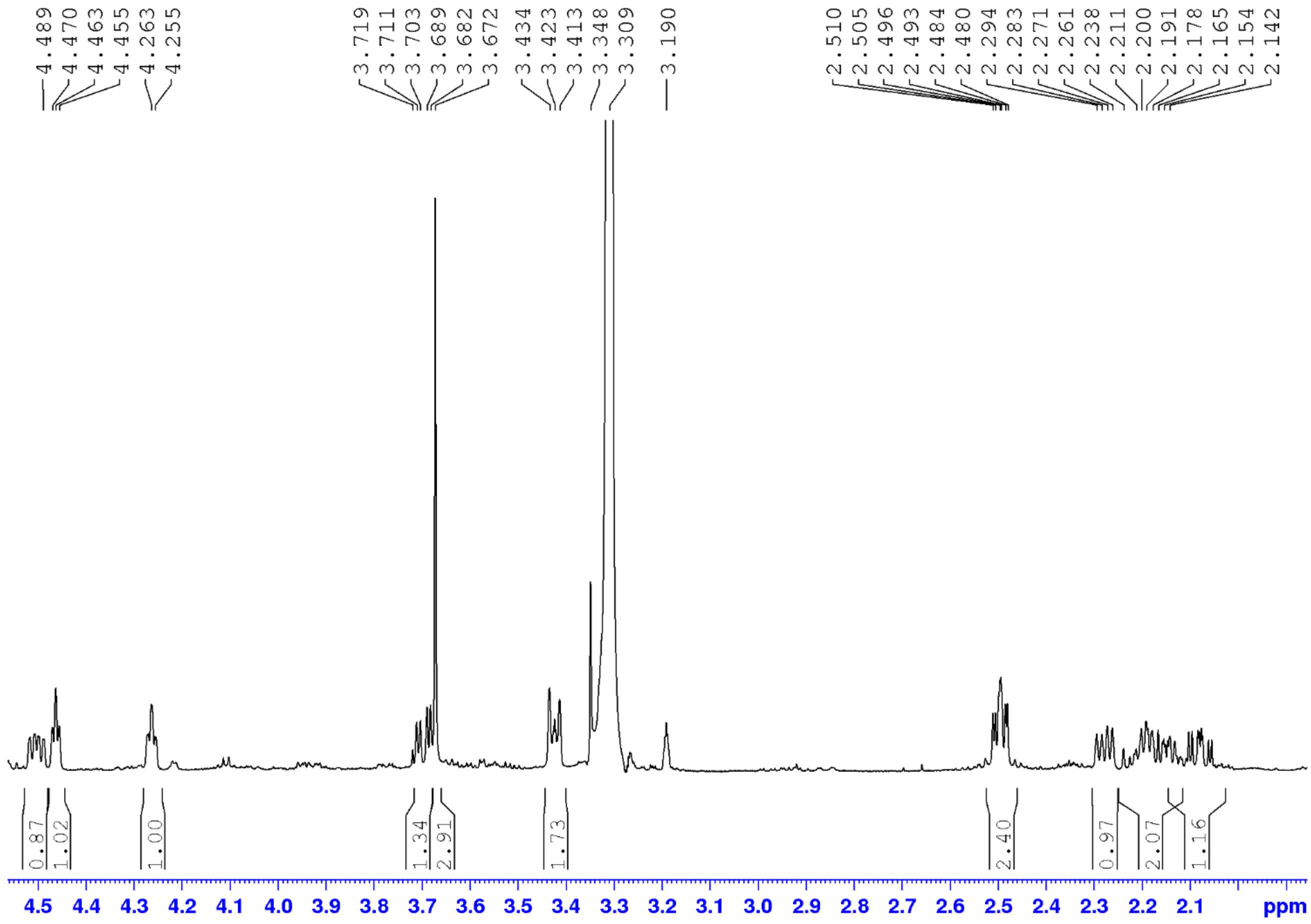

Figure S12. ^{1}H NMR spectrum of cyclo(5-OMe-Glu-4-OH-Pro).

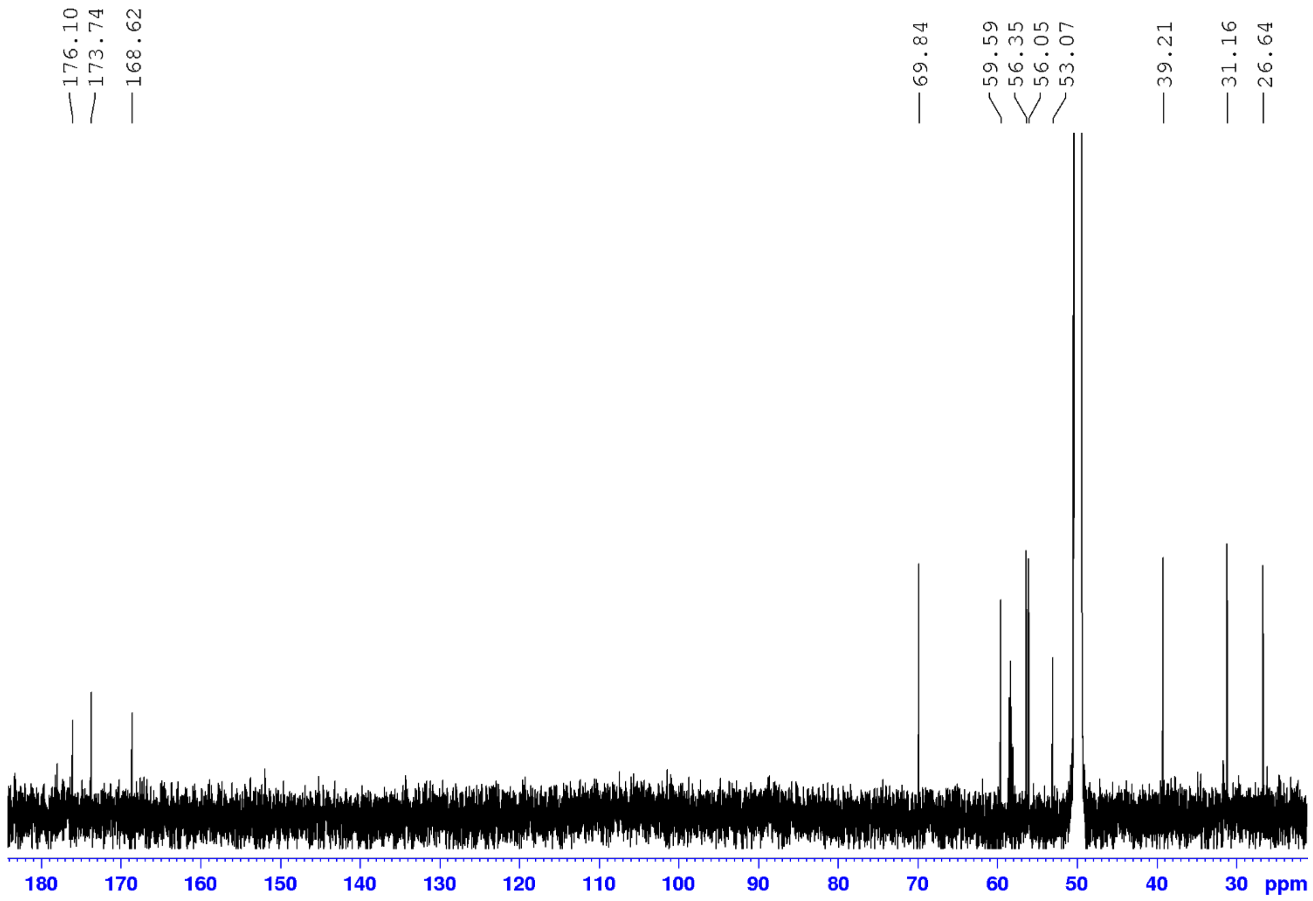

Figure S13. ^{13}C NMR spectrum of cyclo(5-OMe-Glu-4-OH-Pro).

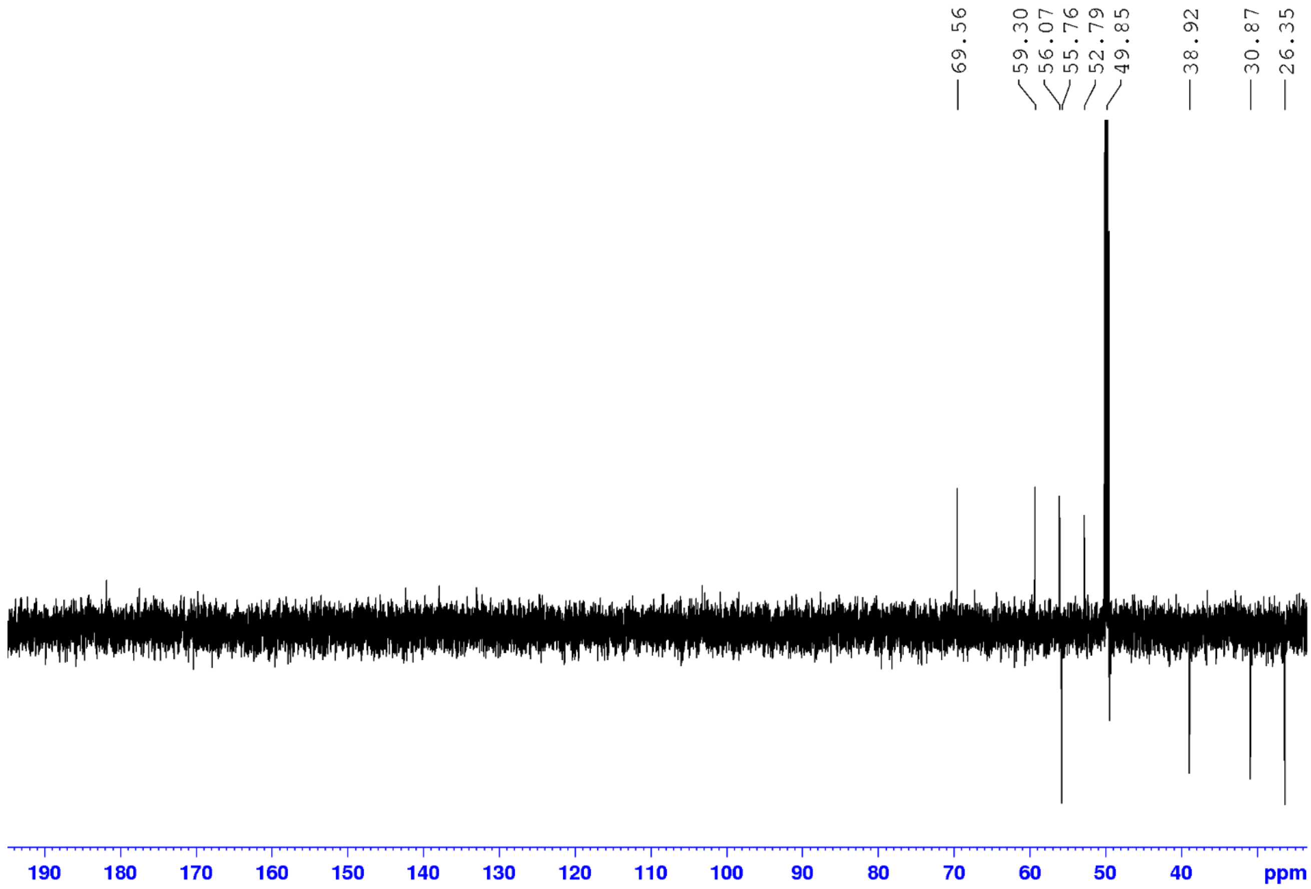

Figure S14. DEPT 135 NMR spectrum of cyclo(5-OMe-Glu-4-OH-Pro).

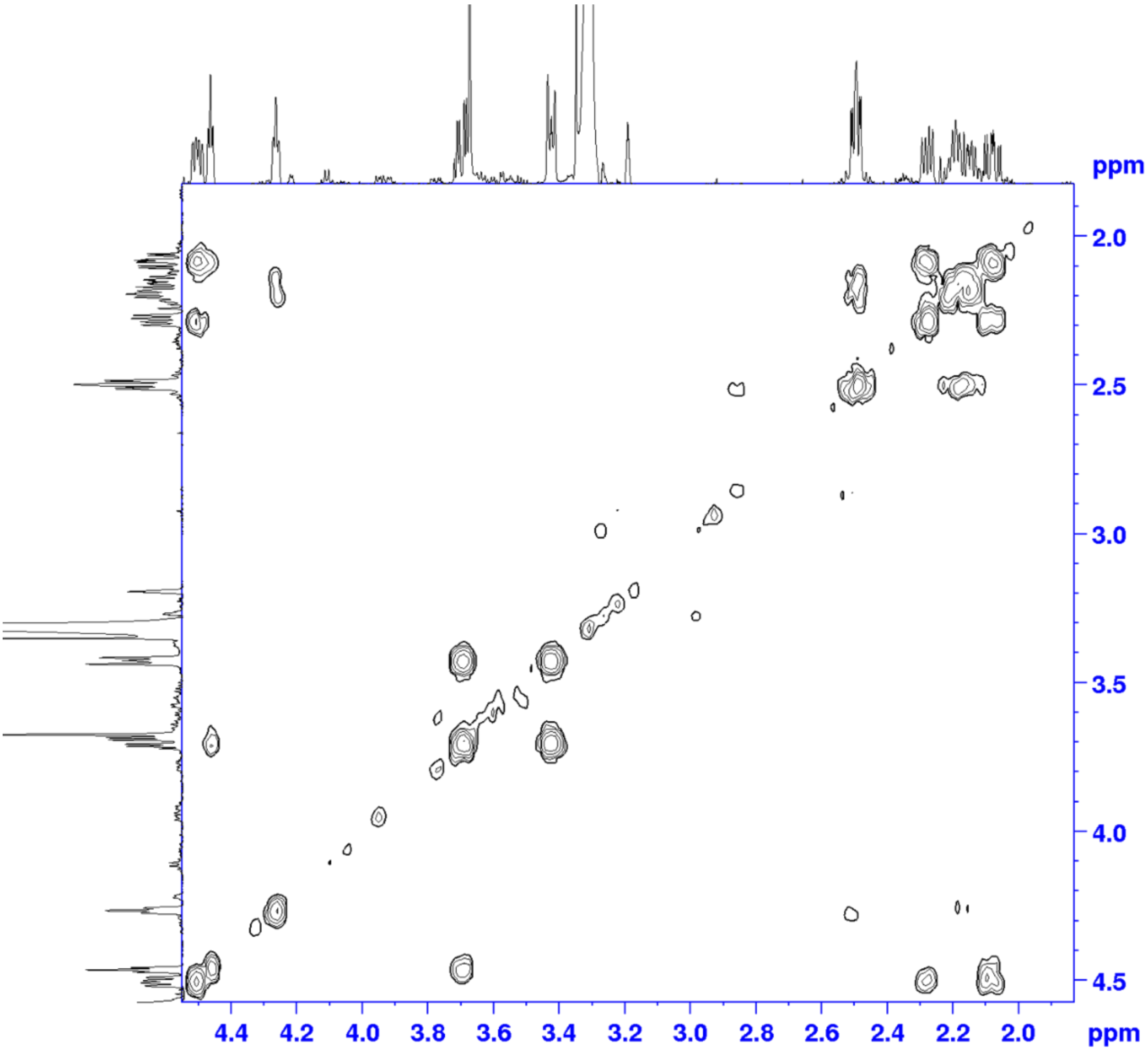

Figure S15. ^{1}H-^{1}H COSY NMR spectrum of cyclo(5-OMe-Glu-4-OH-Pro).

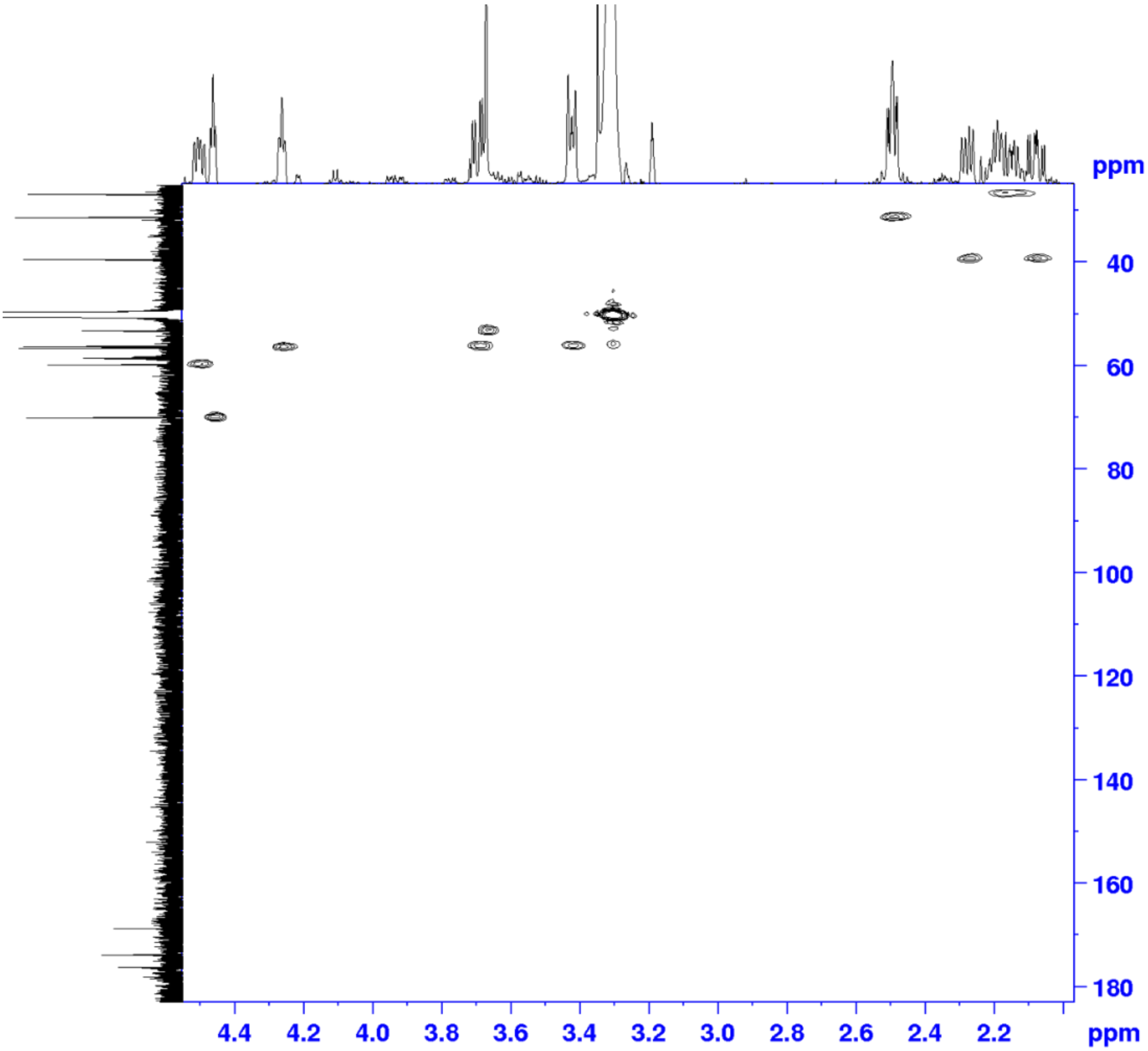

Figure S16. HSQC NMR spectrum of cyclo(5-OMe-Glu-4-OH-Pro).

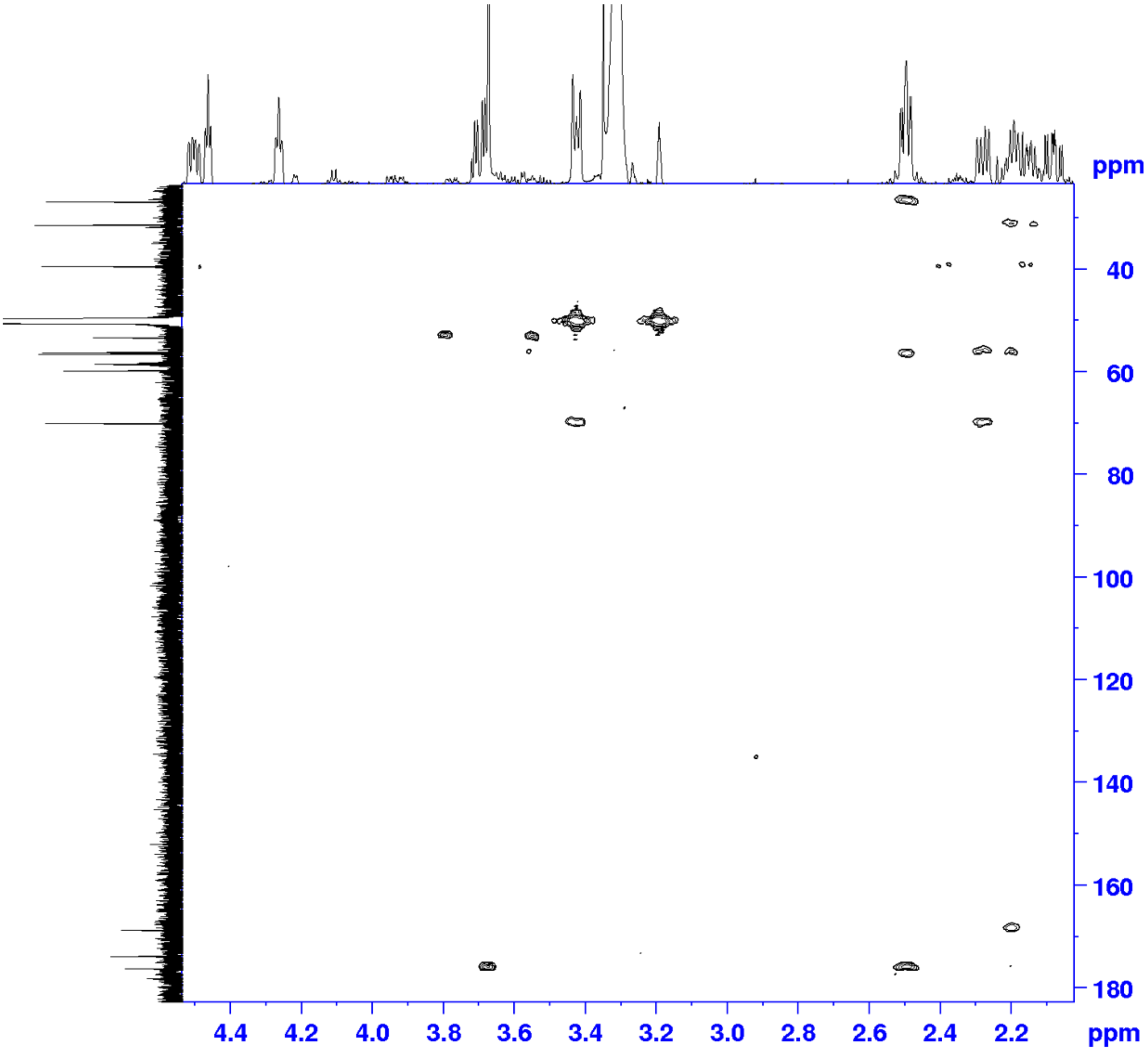

Figure S17. HMBC NMR spectrum of cyclo(5-OMe-Glu-4-OH-Pro).

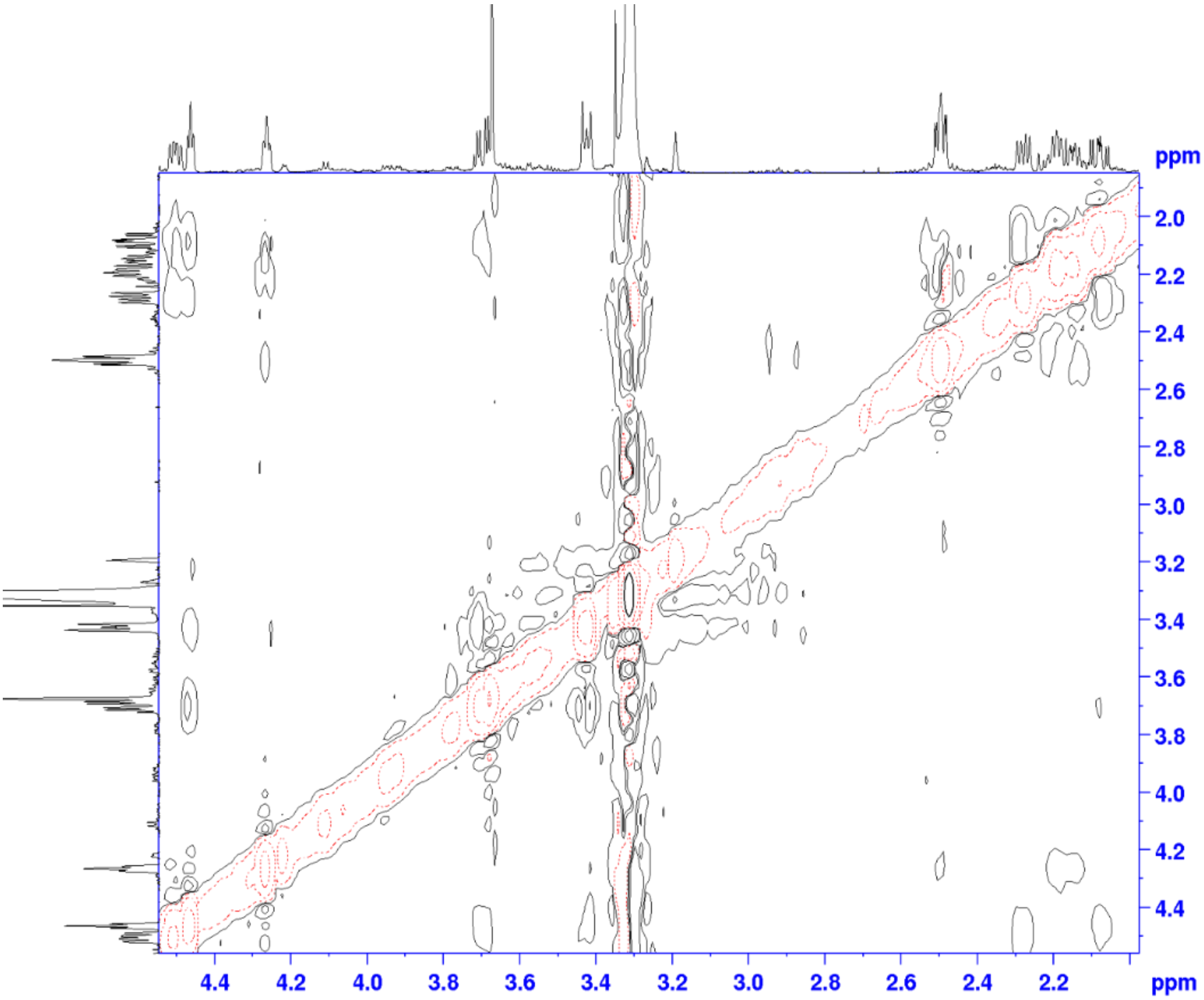

Figure S18. NOESY NMR spectrum of cyclo(5-OMe-Glu-4-OH-Pro).

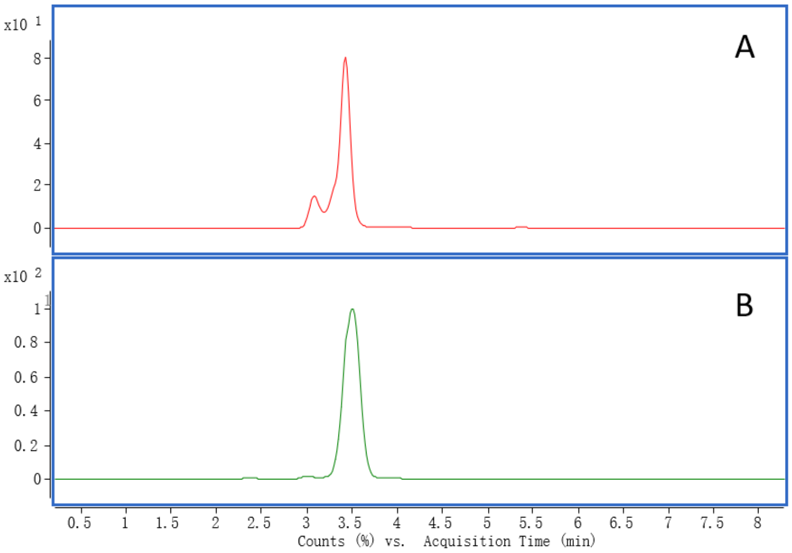

Figure S19. EIC spectra of cyclo(5-OMe-Glu-4-OH-Pro) (m/z = 257.1132) in collected fraction (A) and purified

sample (B).

Table S1. The compounds identified via FBMN. Compound name, *m/z*, and PI/Database found by FBMN.

No.	Compound Name	*m/z*	PI/Database
1	cyclo(Val-Pro) or isomer	197.1286	Keyzers from GNPS-LIBRARY
2	cyclo(Val-Pro) or isomer	197.1286	Keyzers from GNPS-LIBRARY
3	cyclo(Ile-Pro)	211.1441	Massbank
4	cyclo(Leu-Pro) or isomer	211.1436	Keyzers from GNPS-LIBRARY
5	cyclo(Leu-Pro) or isomer	211.1447	Keyzers from GNPS-LIBRARY
6	cyclo(Val-Leu)	213.1594	Pieter Dorrestein from NIST14
7	cyclo(Leu-Hyp) or isomer	227.1385	Keyzers from GNPS-LIBRARY
8	cyclo(Leu-Hyp) or isomer	227.1387	Keyzers from GNPS-LIBRARY
9	cyclo(Phe-Pro)	245.128	Pieter Dorrestein from NIST14
10	cyclo(Phe-Hyp) or isomer	261.123	Keyzers from GNPS-LIBRARY
11	cyclo(Phe-Hyp) or isomer	261.124	Keyzers from GNPS-LIBRARY
12	cyclo(Tyr-Pro)*	261.1237	Jadhav/Dorrestein from NIH-NATURALPRODUCTSLIBRARY
13	Pro-Val*	215.1389	Gabriel Haddad from NIST14
14	Ile-Glu	261.1421	Joshua Wollam from NIST14
15	Leu-Glu*	261.1432	Piel, Dittmann from NIST14
16	Lac-Leu	204.123	Dorrestein from NIST14
17	PyroGlu-Val or isomer	229.1177	Joshua Wollam from NIST14
18	PyroGlu-Val or isomer	229.1179	Joshua Wollam from NIST14
19	PyroGlu-Phe	277.1179	System Wide MS course from NIST14
20	1-Methyl-1,2,3,4-tetrahydro-β-carboline-3-carboxylic acid	231.1144	Trent Northen from BERKELEY-LAB
21	1,3-Diphenylguanidine	212.1182	Massbank

*The compounds co-identified compounds by PEAKS studio.

Table S2. The compounds identified by PEAKS studio. Compound name, *m/z*, and Type of modification found by PEAKS studio.

No.	Compound	*m/z*	Type of modification
1	V	118.0654	—
2	PS(-18.01)**	185.0920	Dehydration
3	PA	187.1086	—
4	P(+72.02)	188.0917	Lactylation
5	AV	189.1235	—
6	T(-18.01)P**	199.1065	Dehydration
7	S(+42.01)(-18.01)A or isomer	201.0869	Acetylation (Protein N-term); Dehydration
8	S(+42.01)(-18.01)A or isomer	201.0873	Acetylation (Protein N-term); Dehydration
9	LA	203.1389	—
10	AL	203.1390	—
11	VA(+14.02)	203.1394	Methylation(C-term)
12	VP	215.1388	—

13	PV*	215.1389	—
14	LA(+14.02)	217.1543	Methylation(C-term)
15	AL(+14.02)	217.1546	Methylation(C-term)
16	PP(+14.02)	227.1383	Methylation(C-term)
17	LP	229.1544	—
18	LT(-18.01)(+14.02)	229.1544	Dehydration; Methylation(C-term)
19	VL#	231.1714	—
20	F(+72.02)	238.1083	Lactylation
21	DL#	247.1302	—
22	P(+27.99)L	257.1490	Formylation
23	PY(-18.01)*	261.1234	Dehydration
24	EL	261.1422	—
25	L(+27.99)T	261.1425	Formylation
26	LE*	261.1432	—
27	VD(+14.02)(+14.02)	261.1436	Methylation(C-term); Methylation(others)
28	FP#	263.1396	—
29	EL(+14.02)	275.1582	Methylation(C-term)
30	E(+14.02)L	275.1590	Methylation(others)
31	VE(+14.02)(+14.02)	275.1591	Methylation(C-term); Methylation(others)
32	T(+42.01)L or isomer	275.1593	Acetylation (N-term)
33	T(+42.01)L or isomer	275.1593	Acetylation (N-term)
34	L(+72.02)A	275.1598	Lactylation
35	AL(+72.02)	275.1600	Lactylation
36	YP	279.1333	—
37	T(+42.01)(+14.02)L	289.1732	Acetylation (Protein N-term); Methylation(others)
38	WT	306.1451	—
39	WS(+14.02)	306.1452	Methylation(C-term)
40	EL(+72.02)	333.1639	Lactylation
41	VGAP#	343.1975	—
42	D(-18.01)APV	383.1920	Dehydration
43	PGLP#	383.2274	—
44	LGVP	385.2447	—
45	LGPV#	385.2463	—
46	D(-18.01)APL or isomer	397.2087	Dehydration
47	D(-18.01)APL or isomer	397.2092	Dehydration
48	LAVP#	399.2597	—
49	LGPL#	399.2606	—
50	VGPQ#	400.2191	—
51	SQPA#	402.2014	—
52	PTPP#	411.2233	—
53	VPPV	411.2600	—
54	PEAP#	413.2021	—
55	VTPP#	413.2435	—
56	LGPAG#	414.2354	—

57	LTPP#	427.2546	—
58	TLPP#	427.2555	—
59	VLPV#	427.2926	—
60	PQSV#	430.2304	—
61	LPPL	439.2895	—
62	LLPP#	439.2921	—
63	LPLP#	439.2931	—
64	LTPL#	443.2856	—
65	APSAL#	458.2648	—
66	PFLP#	473.2768	—
67	PFPL#	473.2773	—
68	LPGVP#	482.2984	—
69	PLGPV#	482.2991	—
70	VTAPP#	484.2766	—
71	TVGLP#	486.2916	—
72	VEPF#	491.2497	—
73	YVPL	491.2850	—
74	VYLP#	491.2850	—
75	VTAAM#	492.2462	—
76	P(+27.99)VSY	493.2305	Formylation
77	PPALP#	494.2988	—
78	RRAP#	499.3115	—
79	RAQF#	521.2823	—
80	RSAPP#	527.2898	—
81	PSGPCA#	531.2235	—
82	PLGGVP	539.3183	—
83	PGLGVP#	539.3190	—
84	SVAAPV#	543.3132	—
85	PLLLP#	552.3749	—
86	LPLLP#	552.3762	—
87	LPLPL#	552.3769	—
88	LPNLP#	553.3336	—
89	FYEP#	555.2432	—
90	VVGVSP#	557.3278	—
91	VVPPF#	558.3295	—
92	SFLVP#	562.3227	—
93	SQAYP#	565.2651	—
94	PFNST#	565.2655	—
95	VVGLSP#	571.3434	—
96	VFDLP#	590.3158	—
97	PLEFP#	602.3179	—
98	VFELP#	604.3391	—
99	RNHSP#	610.3088	—
100	AGPLGVP#	610.357	—

101	VGPTGPV#	626.3524	—
102	PFPGLP#	627.3511	—
103	PPQPLP#	648.3720	—
104	VLPVPQ#	652.4046	—
105	VAPFPE#	659.3394	—
106	RMPPSP#	684.3537	—
107	AVPLTPT#	698.410	—
108	KCKLLP#	701.4369	—
109	YPFPEV#	751.3622	—
110	YPVEPF#	751.3649	—
111	VAPFPEV#	758.4068	—
112	YPFELP#	765.3797	—
113	VAPFPEL#	772.420	—
114	YLTSAQP#	779.3996	—
115	YPFPGPL#	790.4149	—
116	PVPFLPQ#	797.4517	—
117	YPFPALP#	804.4299	—
118	RFDLPLP#	857.4897	—
119	DQFLPYP#	879.4238	—
120	VYPFPGPL#	889.4804	—
121	VAPFPEVFA#	976.5118	—
122	VPPFLQPEV#	1025.5632	—
123	VYPFPGPLPN#	1100.5813	—
124	VYPFPGPLDP#	1101.5594	—
125	VYPFPGPLPQ#	1114.5959	—
126	YVPFPGPLEP#	1115.5733	—
127	VYPFPGPLEP#	1115.5795	—
128	VYPFPGPLPE#	1115.5812	—
129	VVPPFLQPEV#	1124.6333	—
130	PFLVVQLEPP#	1138.6438	—
131	PFLVVVPAADLP#	1237.7152	—
132	VPVVPPFLQPEV#	1320.7523	—
133	TLEQLFPPVLVPVPNTPLP#	2070.1765	—

*The compounds co-identified compounds by FBMN. **Compounds subsequently identified as CDPs. # The compounds enriched by MCX-SPE.

Table S3. ^{1}H NMR (600 MHz, CD_3OD), ^{13}C NMR (150 MHz, CD_3OD) HMBC, ^{1}H-^{1}H COSY and NOESY spectra data of cyclo(5-OMe-Glu-4-OH-Pro).

Position	δ_H (ppm) , mult (J in Hz)	δ_C (ppm)	HSQC	HMBC	^{1}H-^{1}H COSY	NOESY
2	—	168.6	—	H-10	—	—
3	4.26 (1H, t, 5.30)	56.3	C-3	H-11	H-10	H-6, H-8, H-10, H-11
5	—	173.8	—	—	—	—
6	4.50 (1H, ddd, 1.20, 6.20, 11.10)	59.6	C-6	—	H-7	H-3, H-7, H-8
7	2.10 (1H, ddd, 4.30, 11.40, 13.00)	39.2	C-7	H-10	H-6, H-8	H-6, H-8
	2.29 (1H, dd, 6.24, 13.20)					
8	4.47 (1H, t, 4.26)	69.8	C-8	H-7 (**δ** 2.29), H-9 (**δ** 3.43)	H-7, H-9	H-3, H-6, H-7, H-9
9	3.43 (1H, d, 12.60)	56.0	C-9	H-7 (**δ** 2.29), H-10 (**δ** 2.24)	H-7, H-8	H-7, H-8
	3.72 (1H, dd, 4.90, 13.00)					
10	2.17 (1H, m)	26.6	C-10	H-11	H-3, H-11	H-3, H-11
	2.24 (1H, m)					
11	2.50 (1H, d, 2.50)	31.2	C-11	H-10	H-10	H-3, H-10
	2.51 (1H, d, 3.10)					
12	—	176.2	—	H-11, H-14	—	—
14	3.67 (3H, s)	53.1	C-14	—	—	—

Table S4. Sequences of the peptides with their scores generated by AIPpred (scores ≥ 0.342) and PeptideRanker (scores ≥ 0.5).

No.	Peptide	PreAIP	Peptide Ranker
1	TLEQLFPPVLVPVPNTPLP	0.554	0.51
2	LPLLP	0.481	0.75
3	KCKLLP	0.471	0.53
4	PLLLP	0.448	0.72
5	VYPFPGPLPN	0.435	0.79
6	RFDLPLP	0.429	0.84
7	YVPFPGPLEP	0.427	0.58
8	VYPFPGPLEP	0.408	0.66

9	LPLPL	0.384	0.82
10	PLEFP	0.378	0.71
11	YPFELP	0.376	0.80
12	RMPPSP	0.373	0.70
13	VYPFPGPLPQ	0.368	0.69
14	LPNLP	0.354	0.66
15	YPVEPF	0.354	0.63
16	VAPFPEVFA	0.353	0.65
17	LPPL	0.352	0.86
18	VYPFPGPLPE	0.352	0.65
19	YPFPALP	0.350	0.92

Table S5. Docking score of peptides and Rolipram with NEK7 (PDB ID: 2WQN), Cat C (PDB ID: 4CDE), and GSDMD (PDB ID: 5WQT), the TLR4/MD2 complex (PDB ID: 3FXI), TNF-α (PDB ID: 1TNF), IL-6 (PDB ID: 1N26), and IL-1β (PDB ID: 3O4O).

		Binding energy (kcal/mol)						
**No.**	**Ligand**	**NEK7**	**Cat C**	**GSDMD**	**TLR4/MD-2**	**TNF-α**	**IL-6**	**IL-1β**
1	cyclo(Glu-Phe)	-7.6	-7.6	-6.2	-7.4	-6.2	-6.0	-8.8
2	cyclo(Phe-Pro)	-7.5	-7.6	-6.6	-7.0	-7.9	-6.1	-8.5
3	cyclo(Tyr-Hyp)	-7.4	-7.6	-6.8	-7.7	-8.3	-6.0	-8.1
4	cyclo(Phe-Hyp)	-7.3	-8.0	-6.5	-7.9	-7.4	-6.9	-8.3
5	PyroGlu-Phe	-7.2	-7.8	-6.4	-7.0	-5.3	-6.1	-7.5
6	Rolipram	-7.2	-7.7	-6.3	-8.0	-7.8	-6.2	-7.8
7	cyclo(Tyr-Asp)	-7.1	-7.7	-6.6	-7.2	-8.3	-6.4	-7.5
8	cyclo(PyroGlu-Tyr)	-7.0	-7.4	-7.1	-7.0	-6.6	-6.7	-8.0
9	cyclo(Tyr-Pro)	-7.0	-7.2	-6.6	-6.5	-5.8	-5.6	-7.6
10	LPNLP	-7.0	-6.5	-6.5	-7.4	-6.0	-7.0	-9.3
11	VYPFPGPLPQ	-7.0	-7.8	-6.3	-9.7	-6.7	-6.8	-9.4
12	YPFELP	-7.0	-7.1	-6.7	-9.3	-6.1	-7.1	-8.9
13	cyclo(Glu-Tyr)	-6.9	-7.6	-6.4	-6.8	-8.1	-6.2	-7.5

14	WS(+14.02)	-6.8	-6.8	-5.9	-6.2	-5.0	-5.8	-7.7
15	PLLLP	-6.8	-6.5	-7.1	-6.9	-6.6	-6.7	-8.1
16	RMPPSP	-6.8	-6.9	-6.0	-6.7	-5.2	-6.2	-7.5
17	VYPFPGPLPE	-6.7	-6.8	-6.2	-9.6	-6.4	-6.0	-8.7
18	VYPFPGPLPN	-6.7	-6.7	-7.1	-9.1	-7.6	-6.9	-9.5
19	cyclo(MeEGlu-Hyp)	-6.6	-6.8	-5.6	-6.3	-5.2	-5.6	-6.7
20	cyclo(Glu-Leu)	-6.6	-6.5	-5.8	-5.9	-7.0	-5.8	-6.4
21	YPFPALP	-6.6	-7.7	-6.4	-10.4	-7.6	-7.0	-8.8
22	cyclo(Glu-Pro)	-6.5	-6.6	-5.6	-5.7	-5.9	-5.2	-6.6
23	L(+72.02)A	-6.5	-6.2	-5.0	-5.6	-5.0	-4.9	-6.8
24	LPLLP	-6.5	-7.3	-6.8	-7.7	-5.3	-5.5	-8.0
25	cyclo(Glu-Ile)	-6.4	-7.1	-5.3	-6.0	-7.2	-5.2	-6.5
26	cyclo(Val-Leu)	-6.4	-6.6	-5.9	-5.9	-7.0	-5.3	-5.9
27	cyclo(Leu-Pro)	-6.4	-6.4	-5.5	-6.7	-5.0	-5.4	-6.2
28	LPPL	-6.4	-6.3	-6.8	-7.6	-5.2	-6.2	-6.4
29	cyclo(Asn-Pro)	-6.4	-6.5	-5.4	-5.9	-5.2	-5.6	-6.5
30	VYPFPGPLEP	-6.4	-7.1	-6.3	-8.9	-7.1	-7.2	-9.4
31	cyclo(Leu-Hyp)	-6.3	-6.6	-5.8	-5.8	-5.3	-5.2	-6.5
32	cyclo(MeEGlu-Pro)	-6.3	-6.4	-5.9	-6.6	-6.9	-5.1	-6.3
33	cyclo(Val-Val)	-6.3	-6.3	-5.5	-6.1	-6.1	-5.3	-6.5
34	VAPFPEVFA	-6.3	-6.6	-7.7	-8.8	-5.3	-6.7	-8.3
35	YVPL	-6.2	-7.5	-6.7	-7.5	-6.9	-6.5	-8.6
36	cyclo(MeGlu-Ile)	-6.2	-6.6	-5.4	-6.0	-7.0	-4.9	-7.2
37	PLEFP	-6.2	-7.6	-6.5	-6.9	-6.4	-5.3	-8.0
38	TLEQLFPPVLVPVPNTPLP	-6.2	-6.4	-6.0	-7.2	-5.7	-5.3	-7.4
39	YPVEPF	-6.2	-7.7	-7.1	-8.2	-7.4	-5.4	-8.2
40	D(-18.01)APL	-6.1	-7.0	-5.9	-6.0	-5.5	-5.4	-6.2
41	cyclo(Asp-Val)	-6.1	-6.4	-5.5	-6.0	-5.1	-4.9	-6.9
42	cyclo(Ile-Pro)	-6.0	-6.9	-5.6	-5.8	-6.0	-5.2	-6.4

43	cyclo(MeEGlu-Val)	-6.0	-6.4	-5.9	-5.5	-6.6	-5.1	-7.0
44	P(+27.99)VSY	-6.0	-6.2	-6.0	-6.7	-5.3	-5.1	-7.2
45	E(+14.02)L	-6.0	-5.9	-4.9	-5.2	-4.9	-5.1	-5.9
46	cyclo(Asn-Val)	-6.0	-6.5	-5.7	-5.8	-7.2	-5.4	-6.7
47	cyclo(Asn-Leu)	-6.0	-6.6	-5.8	-6.0	-5.2	-5.1	-6.2
48	LPLPL	-6.0	-6.1	-6.0	-7.9	-5.3	-6.3	-7.5
49	YVPFPGPLEP	-6.0	-7.4	-7.0	-9.1	-6.2	-8.1	-8.5
50	LGVP	-5.9	-7.2	-5.9	-6.4	-8.5	-5.2	-6.8
51	T(+42.01)L	-5.9	-6.8	-5.2	-5.2	-5.0	-5.2	-6.0
52	PyroGlu-Val	-5.9	-6.7	-5.4	-6.0	-5.4	-5.4	-6.4
53	P(+27.99)L	-5.9	-6.3	-5.6	-5.5	-6.8	-5.3	-6.4
54	cyclo(Ala-Ile)	-5.9	-6.1	-4.9	-5.5	-6.1	-5.1	-5.6
55	cyclo(Asn-Ile)	-5.9	-6.7	-5.8	-5.7	-6.9	-5.5	-7.2
56	EL(+14.02)	-5.8	-6.3	-5.1	-5.2	-6.3	-5.0	-5.9
57	VP	-5.8	-6.0	-5.4	-4.2	-6.1	-4.8	-5.6
58	cyclo(Ala-Leu)	-5.8	-5.9	-5.4	-5.5	-4.6	-5.1	-6.4
59	EL(+72.02)	-5.7	-6.6	-5.5	-6.2	-5.0	-5.3	-6.8
60	cyclo(Val-Pro)	-5.7	-6.3	-5.8	-5.8	-4.6	-5.2	-6.2
61	AL(+72.02)	-5.7	-6.3	-5.1	-5.7	-7.6	-5	-6.4
62	cyclo(Ser-Leu)	-5.7	-5.9	-4.8	-5.6	-6.1	-5.0	-5.8
63	VPPV	-5.6	-7.6	-5.7	-6.3	-5.2	-5.9	-7.0
64	cyclo(Pro-Pro)	-5.6	-6.6	-5.9	-6.1	-5.0	-5.6	-6.3
65	EL	-5.6	-6.3	-5.3	-5.2	-4.6	-5.2	-6.0
66	L(+27.99)T	-5.6	-5.9	-5.0	-5.5	-4.7	-4.7	-5.9
67	cyclo(Ser-Pro)	-5.6	-5.8	-5.2	-6.0	-6.4	-5.1	-5.8
68	PV	-5.6	-5.8	-5.2	-5.5	-6.6	-5.4	-7.0
69	cyclo(Ala-Val)	-5.6	-5.8	-4.9	-5.6	-6.0	-4.9	-5.4
70	S(+42.01)(-18.01)A	-5.6	-5.8	-4.9	-5.5	-6.0	-4.7	-6.2
71	LT(-18.01)(+14.02)	-5.6	-5.8	-4.9	-5.4	-5.0	-5.1	-5.3

72	PP(+14.02)	-5.5	-6.5	-5.3	-5.4	-6.7	-5.0	-6.3
73	LE	-5.5	-6.5	-5.1	-5.2	-4.7	-4.9	-6.4
74	D(-18.01)APV	-5.5	-6.3	-6.0	-6.6	-5.5	-5.4	-6.9
75	LA(+14.02)	-5.5	-5.4	-4.9	-4.7	-4.4	-4.8	-5.9
76	KCKLLP	-5.5	-5.6	-4.9	-4.7	-4.9	-5.6	-6.9
77	RFDLPLP	-5.5	-6.8	-7.2	-6.1	-5.4	-6.9	-8.1
78	cyclo(Thr-Pro)	-5.4	-6.5	-5.6	-5.2	-7.0	-5.5	-6.0
79	IE	-5.4	-6.5	-5.2	-5.6	-6.8	-5.1	-5.9
80	cyclo(Ala-Hyp)	-5.4	-6.4	-5.1	-6.4	-6.5	-4.9	-7.0
81	cyclo(Asp-Ile)	-5.4	-6.3	-5.7	-5.8	-7.0	-5.5	-6.3
82	cyclo(Ala-Pro)	-5.4	-6.0	-5.2	-5.8	-5.9	-5.1	-6.3
83	LA	-5.4	-5.7	-4.6	-5.0	-6.0	-4.6	-5.8
84	T(+42.01)(+14.02)L	-5.3	-6.3	-5.1	-5.7	-4.4	-4.9	-5.9
85	AV	-5.3	-5.7	-4.9	-5.1	-4.3	-4.6	-5.6
86	AL	-5.3	-5.6	-4.7	-5.1	-4.2	-4.5	-5.3
87	AL(+14.02)	-5.3	-5.4	-4.6	-5.1	-5.6	-4.8	-5.2
88	VD(+14.02)(+14.02)	-5.0	-5.7	-5.0	-5.0	-4.7	-4.8	-5.7
89	VA(+14.02)	-4.9	-5.6	-5.0	-5.5	-4.7	-4.3	-5.5
90	cyclo(Gly-Pro)	-4.9	-5.6	-5.0	-5.4	-5.7	-5.0	-5.4
91	VE(+14.02)(+14.02)	-4.8	-5.8	-5.2	-5.1	-4.5	-5.2	-5.5
